# New Materials for Thin-Film Solid-Phase Microextraction (TF-SPME) and Their Use for Isolation and Preconcentration of Selected Compounds from Aqueous, Biological and Food Matrices

**DOI:** 10.3390/molecules29215025

**Published:** 2024-10-24

**Authors:** Witold Krumplewski, Iwona Rykowska

**Affiliations:** Department of Chemistry, Adam Mickiewicz University, Ul. Uniwersytetu Poznańskiego 8, 61-614 Poznań, Poland; witkru@st.amu.edu.pl

**Keywords:** microextraction, thin-film solid-phase microextraction, thin-film microextraction, extraction coatings, sorption phase

## Abstract

Determination of a broad spectrum of analytes, carried out with analytical instruments in samples with complex matrices, including environmental, biological, and food samples, involves the development of new and selective sorption phases used in microextraction techniques that allow their isolation from the matrix. SPME solid-phase microextraction is compatible with green analytical chemistry among the sample preparation techniques, as it reduces the use of toxic organic solvents to the minimum necessary. Over the past two decades, it has undergone impressive progress, resulting in the development of the thin-film solid-phase microextraction technique, TF-SPME (the thin-film solid-phase microextraction), which is characterized by a much larger surface area of the sorption phase compared to that of the SPME fiber. TF-SPME devices, in the form of a mostly rectangular metal or polymer substrate onto which a thin film of sorption phase is applied, are characterized, among others, by a higher sorption capacity. In comparison with microextraction carried out on SPME fiber, they enable faster microextraction of analytes. The active phase on which analyte sorption occurs can be applied to the substrate through techniques such as dip coating, spin coating, electrospinning, rod coating, and spray coating. The dynamic development of materials chemistry makes it possible to use increasingly advanced materials as selective sorption phases in the TF-SPME technique: polymers, conducting polymers, molecularly imprinted polymers, organometallic frameworks, carbon nanomaterials, aptamers, polymeric ionic liquids, and deep eutectic solvents. Therefore, TF-SPME has been successfully used to prepare analytical samples to determine a broad spectrum of analytes in sample matrices: environmental, biological, and food. The work will be a review of the above-mentioned issues.

## 1. Introduction

Modern instrumental methods of analytical chemistry, including gas chromatography GC and high-performance liquid chromatography HPLC methods, allow the determination of trace amounts of analytes in tiny sample volumes. In analytical procedures for determining micro-trace amounts of analytes, the sample preparation stage plays a crucial role because it involves the separation of analytes from the matrix and their preconcentration to such values that allow analysts to conduct detection or determination. The stage of analytical procedures mentioned above should be consistent with the tenets of sustainable eco-development and the principles of green chemistry, which include the need to reduce the use of toxic organic solvents as much as possible. Compatible with green analytical chemistry, sample preparation techniques include solid-phase microextraction techniques, which are increasingly used in analytical laboratories. Solid-phase microextraction (SPME), developed in the 1990s by J. Pawliszyn, enables rapid, accurate, reproducible, and selective isolation of compounds from a liquid or gas matrix. Over the past two decades, there has been significant development of SPME, which has resulted, among other things, in the development of the thin-film solid-phase microextraction technique, TF-SPME (thin-film solid-phase microextraction). The SPME technique involves the sorption of analytes from a matrix into a solid phase placed on the surface of a thin glass fiber or metal rod. In the TF-SPME technique, the sorption phase is applied to a flat substrate or is a self-supporting membrane. Microextraction sorbents used in the TF-SPME technique are characterized by a larger surface area of the sorption phase than the SPME fiber. Consequently, they have a higher sorption capacity, enabling faster microextraction of analytes than microextraction conducted on the SPME fiber. The dynamic development of materials chemistry makes it possible to use increasingly advanced materials as sorption phases in TF-SPME techniques and to perform selective microextractions from very complex matrices with analytes in trace amounts.

Over the last few years, a large number of reviews, dedicated to solid-phase microextraction, have been published. They present various aspects of the application of solid-phase microextraction techniques in the determination of analytes isolated from complex aqueous [1], biological, and food matrices [2,3,4,5,6]. However, there are few publications of the review type devoted exclusively to the TF-SPME technique. According to the knowledge of the authors of this publication, only three review-type papers are related to the TF-SPME technique. Fatemeh, Pawliszyn, and co-workers discuss the fundamental principle of TFME and its benefits versus the rod fiber geometry of SPME and demonstrate the agreements of the experimental data for the available TFME systems with the theoretical concept [7]. Olcer and co-workers show, in their review, coating techniques for applying sorption layers to solid substrates [8]. In addition to the techniques used in the preparation of TF-SPME devices, Emmons and co-workers describe pivotal steps in the development of the TF-SPME technique with thermal desorption [9]. The main aim of our review is to present the development of the TF-SPME technique over the period 2010–2024. It contains an overview of the most commonly used techniques of applying active phases in the preparation of TF-SPME instruments, the characteristics of materials used as sorption phases, and the characteristics of analytes isolated from water, food, and biological matrices, using the TF-SPME technique.

## 2. Microextraction Techniques

Microextraction techniques (METs) are based on sorption processes in which the volume of the extraction phase is very small compared to the volume of the sample. METs can be divided into techniques based on extraction to the solid-phase SPME (solid-phase microextraction) and to the liquid-phase LPME (liquid-phase microextraction). In LPME liquid-phase microextraction techniques, the extraction system consists of microliters of an immiscible solvent and an aqueous phase containing analytes.

**Figure 1:**
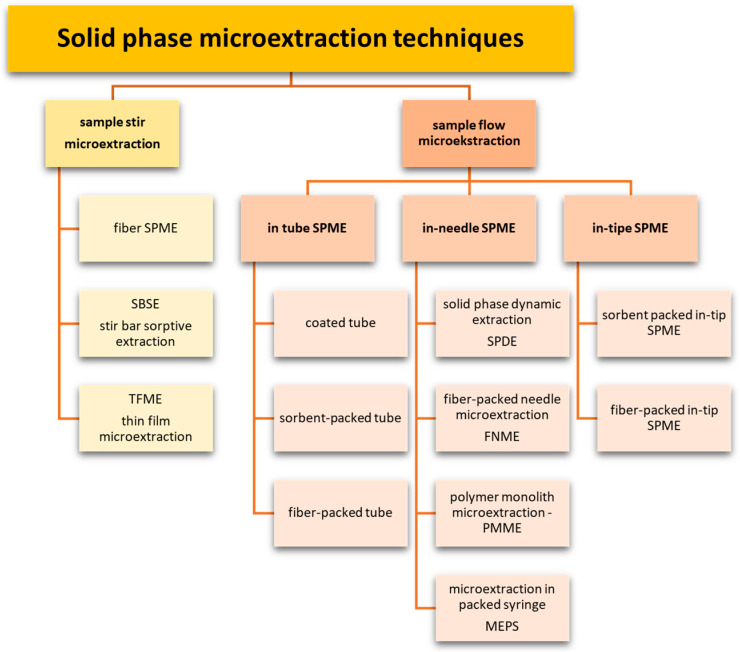
Division of microextraction techniques to the solid phase (SPME).

Solid-phase microextraction methods can be broadly divided into static and dynamic. In the former methods, microextraction is conducted in a closed vessel with a stirrer, and mass transfer of the analyte, from the matrix to the extraction phase, is assisted by stirring (sample stir microextraction). In the latter case, the sample flows through a capillary or a needle with an inside deposited sorptive phase (sample flow microextraction) (Figure 1) [10].

In sample stirred systems, the sorption surface is applied to the following:

1. Thin glass fiber or metal rod (fiber SPME);
2. Rotating magnetic dipole (SBSE);
3. Solid substrate in the form of a thin film (TF-SPME).

In the TF-SPME technique, the polymeric sorption phase can also be a self-supporting membrane.

**Figure 2:**
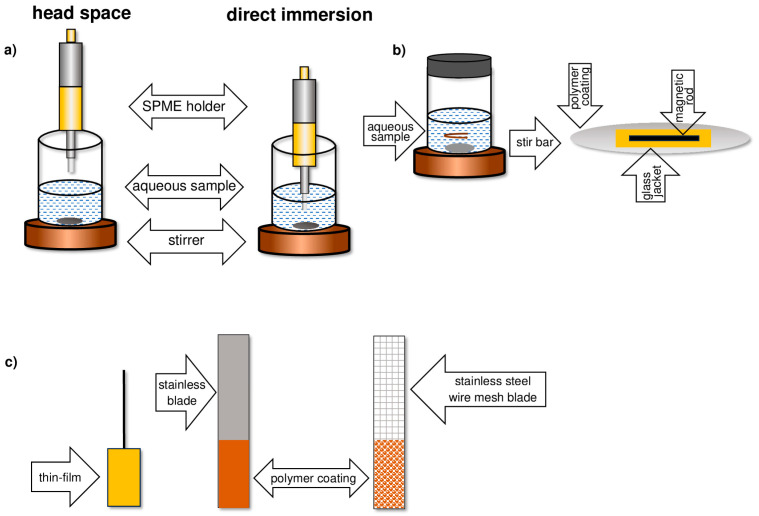
Solid-phase microextraction with sample stirring: (**a**) fiber SPME, (**b**) stir bar sorptive extraction—SBSE, (**c**) thin-film microextraction—TFME.

**Figure 3:**
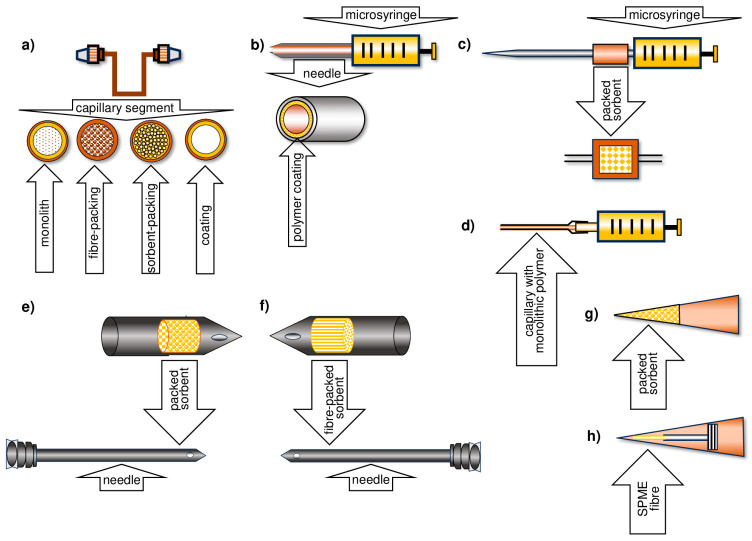
Solid-phase microextraction with sample flow: (**a**) in tube SPME, (**b**) solid-phase dynamic extraction—SPDE, (**c**) microextraction in packed syringe—MEPS, (**d**) polymer monolith microextraction—PMME, (**e**) fiber-packed needle microextraction—FNME, (**f**) microextraction “in needle”, (**g**) sorbent-packed in-tip SPME, (**h**) fiber-packed in-tip SPME.

In sample flow systems, the sorption phase is placed on the surface of a capillary (in-tube SPME), inside a needle (in-needle SPME), or in a micropipette tip (in-tip SPME). Illustrative diagrams of solid-phase microextraction techniques are shown in Figure 2 and Figure 3.

## 3. Solid-Phase Microextraction SPME

SPME microextraction is one of the static methods for isolating and concentrating analytes from solid, liquid, and gaseous samples. It involves the sorption of an analyte in a very thin layer of stationary phase deposited on the surface of a glass or quartz fiber. It was first applied in an analytical procedure in 1990 by J. Pawliszyn [11]. It is now widely used in analytical laboratories in the preparation and testing of environmental [11], food, clinical, and pharmaceutical samples [12] primarily considering:

1. Complete elimination of organic solvents from the analytical workflow;
2. Short time of the analyte extraction step;
3. Simplicity and speed of performing the analysis;
4. The possibility of sampling in situ and in vivo systems [13];
5. High sensitivity of substance determination at the ppt level;
6. An ability to automate the analytical procedure [14];
7. The possibility of desorption of analytes directly in the dispenser of the measuring device.

**Figure 4:**
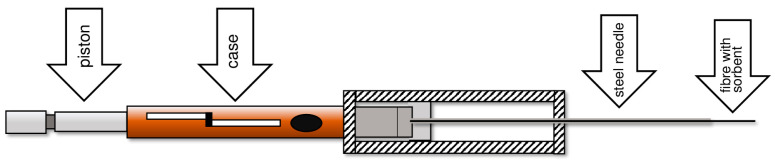
Commercial SPME apparatus construction diagram.

Since 1993, commercial devices have been produced that allow microextraction to be carried out using the SPME technique. They contain a quartz fiber coated either with a polymer film or a polymer layer with a solid sorbent. The fiber is placed in a thin steel tube fixed to a micro-syringe-like holder. When the piston is pressed, the fiber slides out of the steel needle (Figure 4).

The adsorption process takes place on the surface of a fiber that is placed in a vial with the test sample. After the adsorption has been completed, the fiber is retracted into the steel needle of the micro syringe. The adsorbed analytes are thermally desorbed in the dispenser chamber of a gas chromatograph, from which they, together with the carrier gas, are transferred to the crucial chromatography column. Here, the analytes are separated, which is a key step in the process, and, in the chromatograph detector, they are detected.

**Figure 5:**
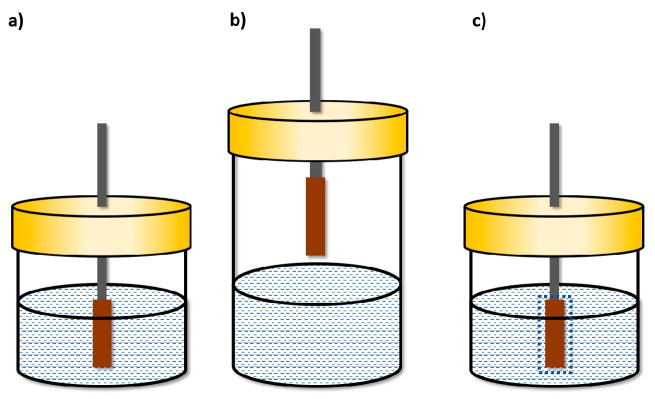
Types of SPME extraction: (**a**) direct extraction, (**b**) superficial extraction, (**c**) protective membrane extraction.

Depending on how the sorptive phase is placed about the sample, the SPME technique can be carried out as follows:

1. In a direct immersion (DI) manner;
2. As adsorption from a headspace (HS) phase;
3. As adsorption with a protective membrane (Figure 5) [15].

In direct DI-SPME microextraction (Figure 5a), the fiber is immersed in the analyzed solution. As a result, analytes are transferred directly from the sample matrix to the sorptive phase of the fiber. In HS-SPME microextraction, relatively volatile analytes that have passed from the liquid phase to the gas phase above the solution surface are adsorbed. During extraction from the headspace (Figure 5b), the fiber is not in direct contact with the solution and, as a result, is not exposed to damage that can be caused by non-volatile impurities present in the sample matrix. However, microextraction with a protective membrane is used for the microextraction of non-volatile compounds from solutions containing interferents with high molecular weights, such as humic acids or proteins (Figure 5c) [16].

The type of organic compound requires selecting an appropriate fiber-coating material to be sorbed since the microextraction efficiency is determined by the affinity of the analytes to the stationary phase. A commonly used group of polymers as extraction phases are silicones, characterized by high thermal and chemical stability and ease of chemical modification. Therefore, these materials enable the selectivity of the sorptive phase to be changed. Extraction fibers coated with polydimethylsiloxane (PDMS), divinylbenzene (DVB), polyacrylate, carboxene (CAR), and polyethylene glycol (PEG/CW) [17] are commercially available.

## 4. Thin-Film Microextraction—TFME

It arises from the theory of microextraction that the total amount of analyte, extracted into a solid phase in a solution in which equilibrium has been established, is directly proportional to the volume of the sorption phase according to the Equation:

(1) $$n=\frac{C_{o}V_{p}V_{s}K}{V_{p}+V_{s}K}$$

in which C_o_ is the concentration of the analyte in the original sample, V_p_—the volume of the sample, V_s_—the volume of the sorption phase (extractant), and K—the equilibrium constant. Thus, with an increase in the thickness of the solid sorption phase, the sorption efficiency increases; at the same time, the sensitivity of the analytical method in which microextraction is used also increases.

Simultaneously, in accordance with the Equation (2) [16]:

(2) $$t_{95\%}=B\cdot \frac{\delta Kd}{D_{f}}$$

where the time in which a state of equilibrium is established in the sorption system is prolonged with the increase in the thickness of the extraction phase. In Equation (2), B—denotes the coefficient related to the geometry of the sorption surface, δ—the thickness of the boundary layer, d—the thickness of the sorption surface, K—the equilibrium constant (the partition coefficient of the analyte between the sorption phase and the sample), and D_f_—the diffusion coefficient of the analyte in the sample.

The rate of solid-phase microextraction is determined by the Equation (3) [18]:

(3) $$\frac{dn}{dt}=\left(\frac{D_{f}A}{\delta }\right)C_{o}$$

is directly proportional to the surface area of the extraction phase. In Equation (3), D_f_—denotes the diffusion coefficient of the analyte in the sample, A—the sorption area, δ—the thickness of the boundary layer, and C_o_—the initial concentration of the analyte in the sample. As a result, the geometry of the extraction phase, in the form of a thin film, for which the surface-to-volume ratio is large, causes an increase in the following:

1. The rate at which the system reaches equilibrium;
2. The sorption capacity of the solid phase.

Hence, the use of a microextraction device with a developed sorption surface increases the speed of sample execution, and it is also a suitable solution for trace analysis [9].

## 5. Development of the TF-SPME Technique

In 2001, Wilcockson and collaborators conceived the first microextraction system, which possessed a solid extraction phase with a higher surface-to-volume ratio than a traditional SPME device. It consisted of a thin film of ethylene-vinyl acetate as the extraction phase, which was applied to glass disks with a diameter of 22 mm [19].

In comparison to 100 μm SPME fiber, the disk had the following:

1. More than 1000 times larger surface-area-to-volume ratio of the sorption phase;
2. A smaller volume of the extraction phase.

As a result, Wilcockson’s microextraction solution significantly reduced the time the system reached equilibrium but did not increase the sensitivity of the analytical method [9].

**Figure 6:**
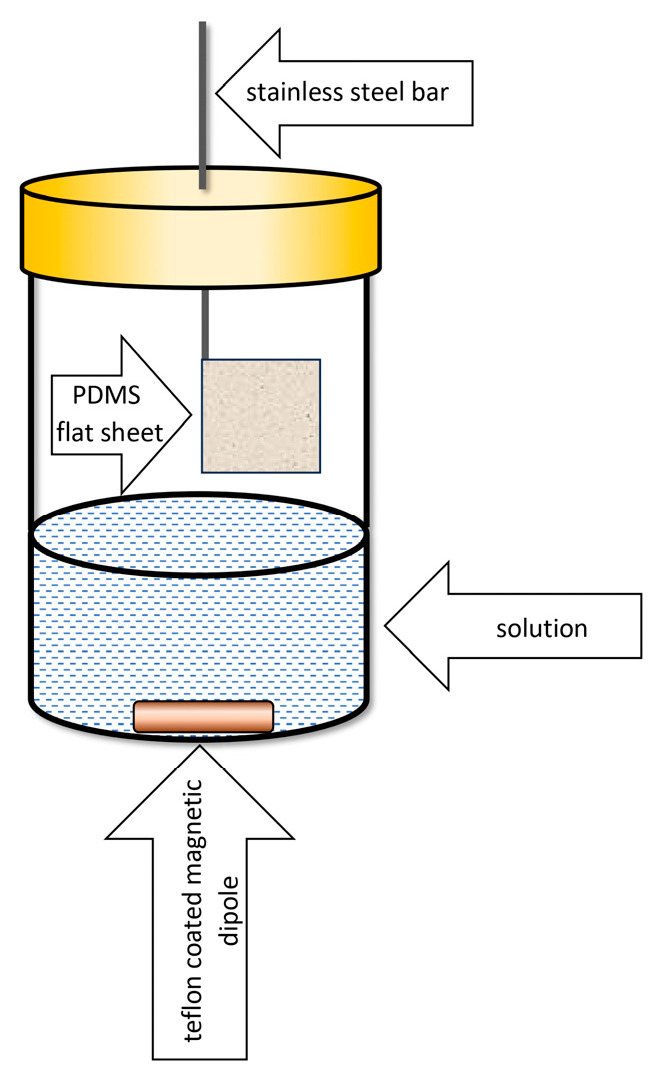
Bruheim TF-SPME system during sorption [18].

In 2003, Bruheim and colleagues developed a microextraction device that contained a 25.4 μm thick membrane of prefabricated PDMS as the extraction phase attached to a stainless-steel rod [18]. The sorption process occurred on an unrolled PDMS sheet, which, together with a metal support, resembled a flag (Figure 6).

After the sorption was completed, the PDMS membrane was rolled up and placed in the thermal desorption unit of the gas chromatograph. In comparison to SPME microextraction performed with a fiber-coated glass with a 100 μm PDMS, a 20-fold higher extraction efficiency of polycyclic aromatic hydrocarbons, extracted from an aqueous matrix, was obtained for a 1 cm × 1 cm PDMS membrane [18].

**Figure 7:**
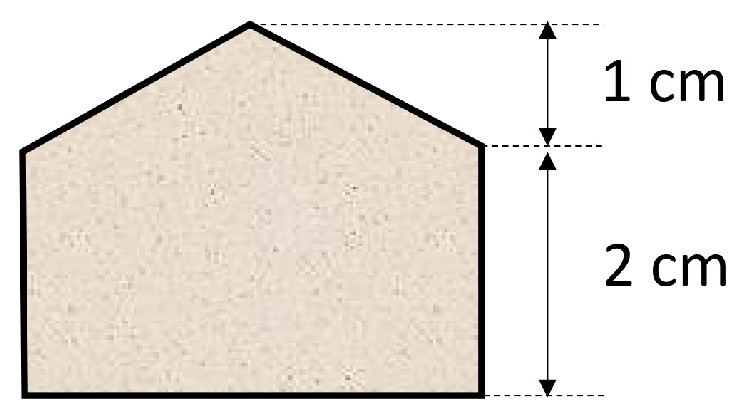
Bragg’s membrane TF-SPME [20].

In 2006, Bragg and co-workers used a 127 μm thick PDMS sheet in microextraction in a 2 cm × 2 cm rectangular shape with an attached 1 cm high triangle hooked to a stainless-steel wire [20] (Figure 7).

The applied modification of the shape of the extraction phase determined the increase in its surface area and its volume compared to the rectangular shape used by Bruheim; so, the sensitivity of the determination of analytes increased. At the same time, the “house shape” of the PDMS sheet allowed it to be rolled around a metal carrier and conveniently placed in the thermal desorption unit of a gas chromatograph.

In the same year, Rodil and his co-workers used for the first time a composite obtained from a combination of polyacrylate and glass wool [21] for the construction of a TF-SPME device. To obtain the microextraction device, they placed the fabric on polyethylene film, which was then saturated with polyacrylate solution and covered with the second sheet of film. After removing the foil layers, they cut the obtained material into strips with dimensions of 6 cm × 0.3 cm, adapted to the dimensions of the thermal desorption unit of the chromatograph. The obtained polyacrylate strips were characterized by significantly higher mechanical stability compared to membranes made of pure PDMS. However, after repeated use, they underwent thermal decomposition resulting in reduced mechanical strength and chromatograph defilement [9].

**Figure 8:**
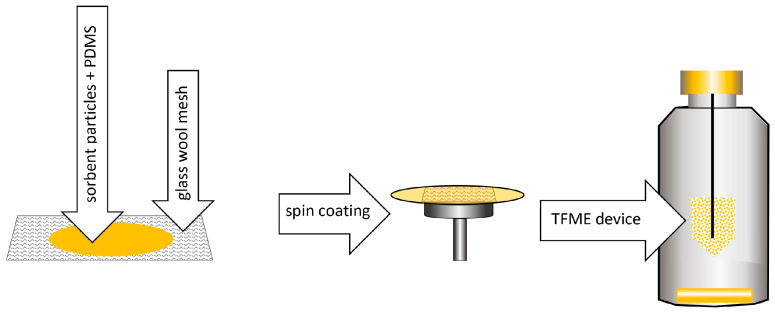
Diagram of the sorption process using the Riazi TF-SPME device [22].

Six years later, Riazi and colleagues developed a TF-SPME device with an extraction phase composed of two components: polydimethylsiloxane and divinylbenzene (PDMS/DVB) or carboxene and divinylbenzene (Car/PDMS) [22]. Adsorbent particles (carboxene or DVB) were dispersed in PDMS solution, then applied to a thin sheet of glass wool mesh and spread by spin coating. The resulting material was cut into a “house shape”—the rectangle measuring 2 cm per 2 cm—and attached to a 1 cm high triangle, which was then linked to a metal pin. Ultimately, the material was placed in an extraction vessel. After sorption was completed, the active surface was rolled, and thermal desorption was carried out (Figure 8).

During the desorption process, the developed sorption material showed significantly higher mechanical and thermal stability compared to Rodil’s strips [22].

In 2013, Jiang and J. Pawliszyn made a PDMS membrane with applied divinylbenzene with a rod-coating method, which was used for the adsorption and determination of benzene in air [23]. PDMS was added to divinylbenzene dispersed in hexane, and then, after sufficiently long sonification, a drop of the resulting mixture was taken with a pipette and applied to aluminum foil. After being mechanically formed, the membrane was vacuum-dried at 120 °C. It was determined that the use of a twenty-percent weight addition of divinylbenzene to PDMS was the optimal ratio to obtain a membrane with good mechanical strength. Compared to the use of SPME fiber or a single-phase PDMS membrane, the use of the DVB/PDMS membrane, obtained by rod coating, determined the increase in the sensitivity of benzene determination in the gas matrix.

In 2016, Grandy and co-workers used a carbon mesh to develop a TF-SPME device, onto which the extraction phase was applied in the form of dispersed DVB in PDMS [24]. The polymer-coated carbon support was cut into rectangular strips with a width determined by the dimensions of the TD unit of the gas chromatograph. The obtained micro-extraction device allowed extraction with high efficiency and had higher mechanical strength compared to previous technical solutions in the TF-SPME method. The carbon mesh strip, coated with a polymer layer as an extraction phase, became part of the first commercial microextraction kit offered by GERSTEL Inc. (Gerstel GmbH, Mulheim, Germany).

The technique of microextraction on a developed surface has not just remained a theoretical concept. It has been successfully applied to a high-throughput automated analytical procedure using robotic autosamplers. In 2009, J. Pawliszyn’s team presented a solution for simultaneous microextraction of 96 samples. The developed TF-SPME device, with the extraction phase as C18 silica applied to stainless steel plates, has proven its practicality. The silica-coated plates were combined into twelve-element batteries and then into an array containing ninety-six elements, which were, among others, used in the analysis of benzodiazepines in urine [25]. This practical application underscores the real-world relevance of our research and its potential to revolutionize analytical procedures.

In recent years, the development of the TF-SPME method has manifested itself in the use of a variety of advanced materials as increasingly selective sorption phases that enable microextraction to be carried out with high efficiency.

## 6. Methods for Obtaining Active Coatings in TFME

**Figure 9:**
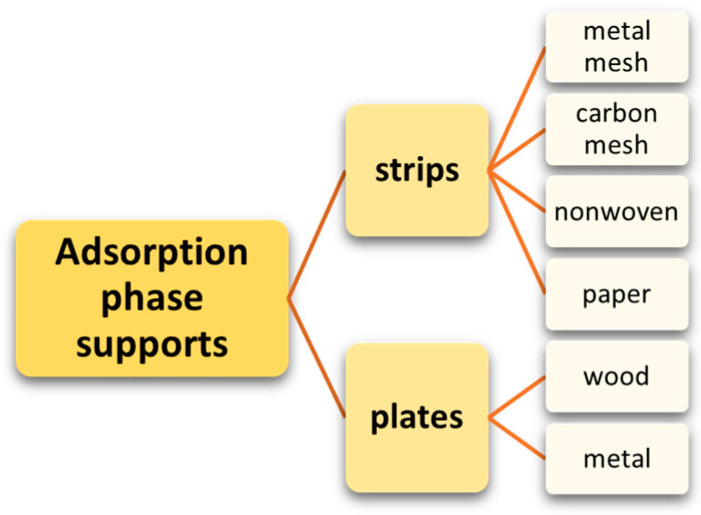
Types of sorptive phase supports in TF-SPME devices.

The choice of the type of extraction phase and the method of its application to the substrate in the process of constructing a TF-SPME device is determined by the chemical properties of the analyte, the type of matrix from which it is sorbed, and the type of desorption method [9]. Thermal desorption requires resistance to high temperatures, while desorption in solvent demands adequate mechanical strength. Consequently, materials with different mechanical and thermal properties are used as extraction-phase supports (Figure 9).

The following techniques for applying thin-film coatings to solid substrates are used: dip coating, spin coating, electrospinning, rod coating, and spray coating.

**Figure 10:**
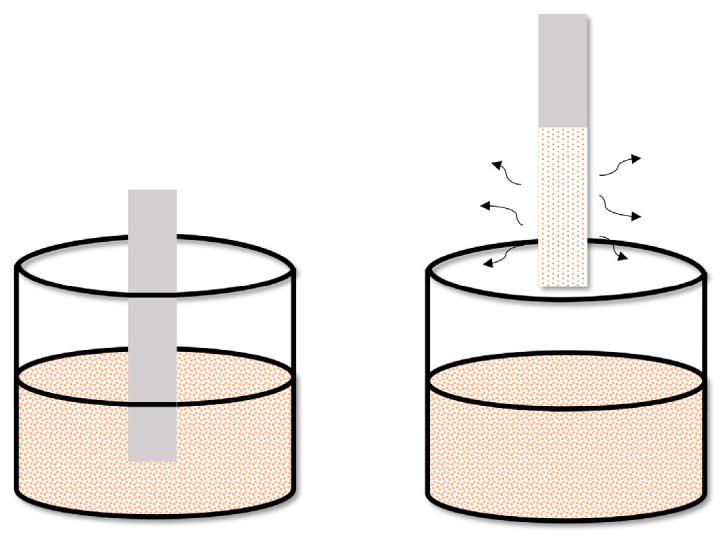
Schematic diagram of the dip-coating method [8].

According to the dip-coating method, a suspension of the extraction phase in a suitable solvent or adhesive is prepared. The polymer or metal substrate is then immersed in it. The thickness of the coating of the active phase is controlled by the rate at which the substrate is removed from the solution and the number of repetitions of the above-mentioned operation (Figure 10).

The dip-coating method is one of the technically more straightforward methods of applying an extraction layer to a solid substrate and, at the same time, the most convenient method for obtaining a homogeneous, very thin active layer with a thickness of a few micrometers. This method is used, among others, in the construction of TF-SPME devices dedicated to detecting analytes by mass spectrometry, in which a short desorption time is crucial because it depends on the thickness of the sorption layer [8].

**Figure 11:**
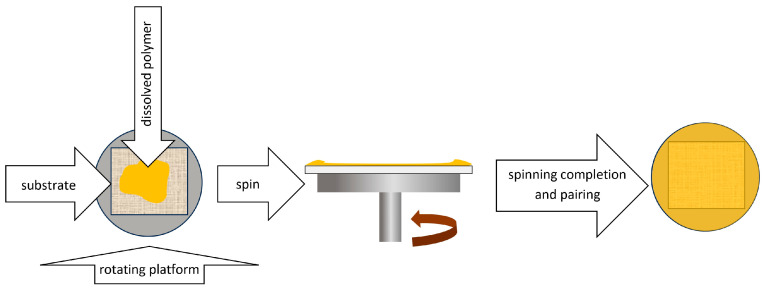
Schematic diagram of the spin-coating method [8].

Spin coating is a mechanical method of applying a sorption layer to a solid substrate, making it possible to obtain a self-supporting TF-SPME membrane. The extraction phase in liquid form is placed on a disk, which is then set in rotary motion at high angular speed (Figure 11).

As a result of centrifugal force, the suspension particles move toward the edge of the substrate, forming a thin polymer layer on its surface. Its thickness is controlled by the rotational speed of the disk [9]. Using the spin-coating method, it is possible to apply several layers that differ in chemical composition. One of the main limitations of the vortex-coating method is the difficulty in obtaining a sorption surface of uniform thickness in both its central and outer parts [8].

**Figure 12:**
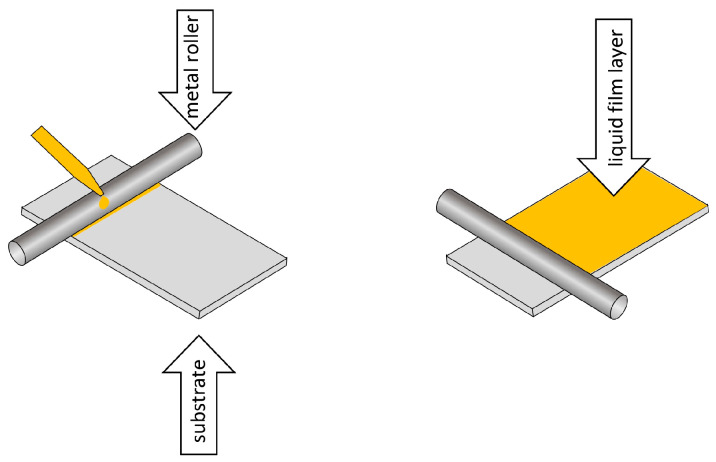
Schematic diagram of the bar-coating method [26].

The first thermally stable polydimethylsiloxane membranes, thermally desorbed and used in TF-SPME, were made using spin-coating and rod-coating methods. Both of the above-mentioned methods make it possible to prepare a TFME device on a support or as a self-supporting membrane. In the rod-coating method, the extraction phase in liquid form is placed on a substrate, such as a film, and then spread using a rod-shaped cylinder to obtain a thin and uniform coating (Figure 12).

The thickness of the extraction layer is controlled by the size of the clearance between the substrate and the mechanical liquid-polymer-spreading element, and by the force of its pressure on the substrate [8].

**Figure 13:**
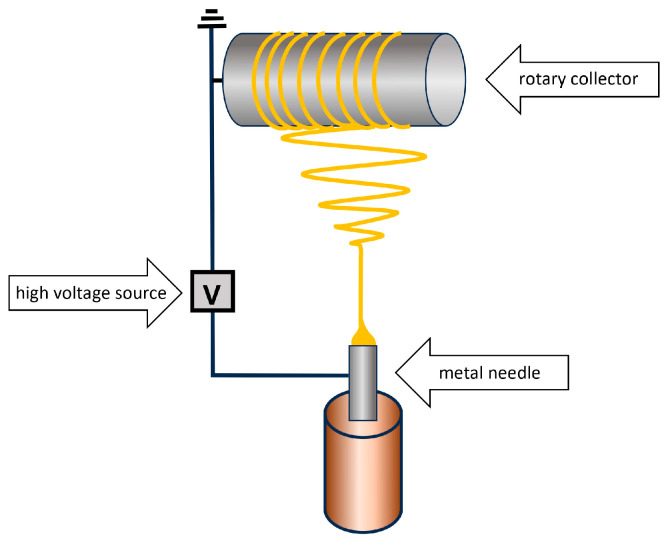
Schematic diagram of the electrospinning-coating method [27].

The electrospinning-coating method uses an electric field to pull electrically charged polymer threads from a polymer solution or polymer melt. The aim of the procedure is to produce mechanically stable membranes with nanofibrous structures [8]. The apparatus, enabling electrospinning, contains a high-voltage generator, a pump, a syringe ending in a capillary, and a conductive collector. The collector can have a flat or oval shape (Figure 13).

Free electric charges appear in a polymer solution placed in a syringe. They flow through an electrode that is immersed in the polymer solution and connected to a high-voltage generator. They move in the generated electric field toward the electrode of opposite polarity. The movement of ionized polymer particles causes a conical deformation in the droplet. When the electric potential, applied to the system, reaches the value required to overcome the surface tension of the liquid polymer, a thin stream of liquid is emitted from the conical end of the droplet. As the liquid polymer jet travels through the electric field, created between the syringe needle and the collector, solvent evaporation occurs. Consequently, a dry polymer fiber is placed on the collector [28]. The thickness of the polymer membrane, formed on the surface of the collector, is under your complete control, determined by the duration of the process.

**Figure 14:**
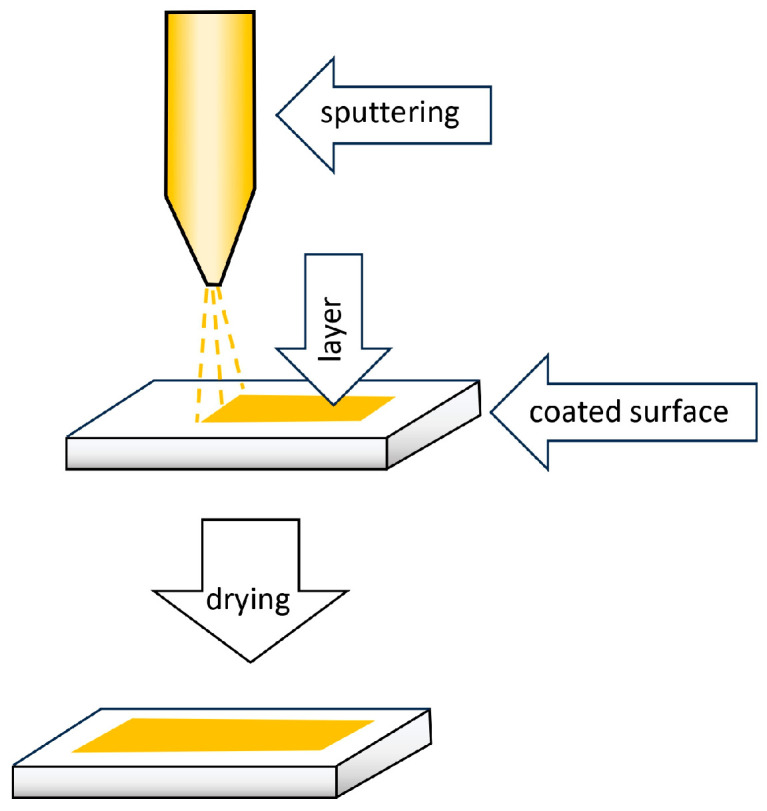
Schematic diagram of the spray-coating method [29].

Spray coating, along with dip coating, is one of the simplest methods of preparing TF-SPME devices. The extraction phase, dissolved or dispersed in a suitable solvent, is placed in the tank of the spray instrument and then pressurized with an inert gas. In the next step, the suspension is sprayed onto a solid support in the form of microscopic droplets (Figure 14).

The method’s disadvantage is the difficulty in obtaining a coating of uniform thickness. This problem can be partially solved by spraying several layers. However, this solution is not perfect. It often causes a coating with a significant thickness, calculated in millimeters, to appear instead of a desirable uniformly thick layer [8].

The advantages and disadvantages of the TF-SPME device preparation methods, outlined above, are shown in Table 1.

## 7. Sorbents Used in the TFME Technique

The variety of physicochemical properties of analytes in samples with complex matrices such as food, biological, and environmental samples requires the use of extraction phases in the TF-SPME technique, which are characterized by sufficient high sensitivity, selectivity, sorption capacity, and mechanical, thermal, and chemical resistance [15]. The K_eq_ partition constant of the sorption surface and the analyte extracted from a given matrix directly impact its microextraction process’s selectivity, efficiency, and speed. The value of the partition constant is determined mainly by the chemical and physical structure of the extraction medium used in the TF-SPME device. The selection of the material constituting the extraction phase is, therefore, a key factor affecting the method’s sensitivity for determining the analyte [30]. Polar sorbents have an affinity to hydrophilic compounds whose molecules contain polar functional groups such as hydroxyl, amine, and carboxyl. A principal feature of non-polar sorbents is a high affinity to hydrophobic organic compounds whose molecules are mainly composed of non-polar hydrocarbon chains or rings.

**Figure 15:**
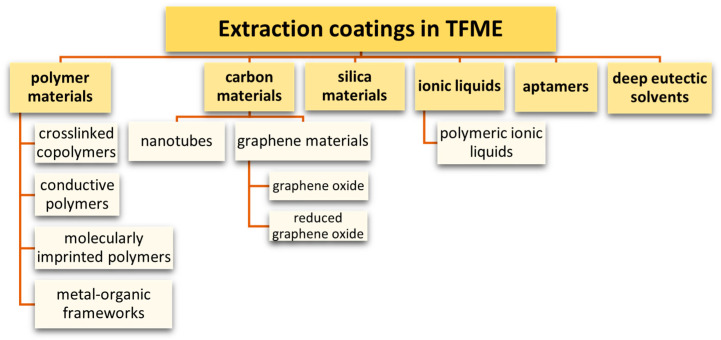
Types of materials used as extraction phases in the TFME technique.

The achievements of recent years in the field of materials chemistry have contributed to the use of the most diverse classes of materials in microextraction techniques. The materials show features such as high specific surface area, good wettability and ease of their chemical modification, and the presence of suitable functional groups that enhance interactions with the analyte [31]. Once they have been used as sorption media in microextraction devices, the characteristics of modern materials permit microextraction with high selectivity and sensitivity to be carried out. In the TF-SPME technique, advanced polymeric materials, carbon nanomaterials, aptamers, and poly (ionic liquids) are used as extraction media (Figure 15).

### 7.1. Polymeric Adsorbents

In TF-SPME, polymers are used as sorptive phases in the form of self-supporting TFME membranes and as sorptive phase dispersion media or as a glue that attaches the solid sorptive phase to the support.

**Figure 16:**
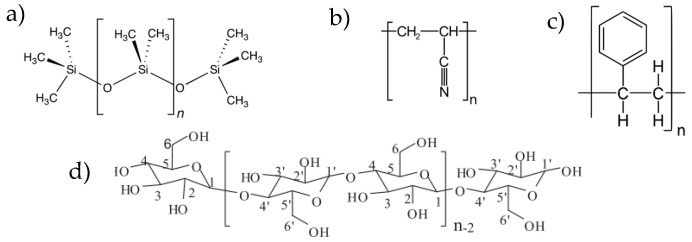
Structural formulas: (**a**) polydimethylsiloxane, (**b**) polyacrylonitrile, (**c**) polystyrene, (**d**) cellulose.

A developed internal surface and the presence of various types of functional groups characterize polymeric adsorbents, which are often cross-linked copolymers. Non-specific dispersive interactions mainly occur between the macromolecules of the polymer and analyte molecules. Various types of specific interactions can also occur, which are related to the presence of functional groups in the polymer macromolecule. Specific interactions allow the formation of hydrogen or ionic bonds between analyte particles and polymer macromolecules. Polymers frequently used in the TF-SPME technique include PDMS, polyacrylonitrile (PAN), polystyrene (PS), and the natural polymer cellulose (Figure 16).

PDMS belongs to the group of first polymers used in the TFME technique. Polydimethylsiloxane is a polymer classified as siloxane. Its macromolecule contains Si-O-Si silicon bridges in its structure, in which each silicon atom is connected to methyl groups (Figure 16a).

PDMS is commonly used as a sorbent in solid-phase microextraction techniques because of its properties, which include the following:

1. High thermal resistance;
2. The ease with which it undergoes desorption;
3. Chemical and physiological inertness;
4. Flexibility and mechanical strength;
5. Low chemical reactivity.

In addition, PDMS is resistant to matrix effects. Its sorption properties do not change even in highly complex matrices such as plasma, animal adipose tissues, or digestive juices [30].

Despite its many positive features, PDMS, as an extraction phase, also has its limitations. It shows low selectivity toward apolar compounds; thus, analyzing samples with complex matrices becomes difficult when used as an extraction phase. As an apolar material, it shows a low affinity to polar analytes. In order to increase the microextraction efficiency of polar analytes, combinations of polydimethylsiloxane with other sorbents, including divinylbenzene (PDMS/DVB) and carboxene (PDMS/CAR), are currently applied in TF-SPME techniques. TFME membranes, made of PDMS-coated carbon mesh and DVB/PDMS or CAR/PDMS, are also found in commercially produced TFME microextraction kits by Markes International, among others.

Polyacrylonitrile (PAN) is a synthetic resin formed by the polymerization of acrylonitrile (Figure 16b). It belongs to the hard and rigid thermoplastic materials. Due to the presence of highly polar nitrile (-CN) groups in the macromolecule, it does not dissolve in typical organic solvents. In the TF-SPME technique, it mainly performs the function either of a dispersion medium, or of a linker of the sorption phase to the substrate.

Polystyrene (PS) is a polymer of the thermoplastic group (with limited elasticity) obtained by polymerization of styrene (Figure 16c). It is soluble in aromatic and chlorinated hydrocarbons and in ketones. It does not dissolve in aliphatic hydrocarbons, alcohols, and water. In microextraction to the solid phase, it is used as both a sorption phase and a dispersant.

Cellulose is a natural polymer commonly found in nature. It is a component of plant cell walls. It belongs to natural raw materials extracted mainly for industrial purposes from wood and cotton. Cellulose products are environmentally safe as they decompose through a natural carbon cycle [32]. The polymer chain of cellulose has a simple, unbranched structure consisting of glucose rings linked by β-1,4-glycosidic bonds (Figure 16d). In view of the absence of hydrocarbon side chains, the cellulose chains form ordered and fibrous semi-crystalline structures. The -OH groups, bonded to carbon atoms, determine the chemical and physical properties of cellulose. Hydrogen bonds are formed between the oxygen and hydrogen atoms of the various hydroxyl groups belonging to other polymer chains. The network of hydrogen bonds makes cellulose insoluble in water and most organic solvents [33]. The fibrous structure of cellulose, which contributes to enlarging the specific surface area, good mechanical stability, ease of chemical modification, and environmental safety, makes it a sorption material with great potential for applications in microextraction techniques. In TF-SPME, cellulose paper is used as a support for the actual sorbent and a component of the sorption phase. It is often subjected to modification with polymeric ionic liquids or other polymers, e.g., polyaniline, polyamide, polydopamine, and zinc oxide or silver nanoparticles.

Examples of the use of the above-described polymers as extraction phases in the TF-SPME technique, used in the analysis of environmental, biological, and food samples and reported in scientific publications, are summarized in Table 2 and Table 3.

### 7.2. Conductive Polymers

Conductive polymers (CPs) are among the polymeric materials that exhibit electrical conductivity along their long molecular chains because they contain conjugated π-electron backbones in the macromolecule. With regard to their electrical, mechanical, thermal, and optical properties, CPs find applications in many fields of science and technology. They are often used as electromagnetic screens, corrosion inhibitors, and electroluminescent displays [48]. The large specific surface area and excellent chemical, mechanical, and thermal stability also make CP materials suitable sorbents often applied in microextraction techniques, including TF-SPME [49].

Polymers showing conductivity are, for example:

1. Polyacetylene (PA) and polyphenylacetylene (PPA), containing double bonds;
2. Polyfluorene (PF) and polyparaphenylene (PPP), polyaniline (PANI), and polyphenylenevinylene (PPV), containing aromatic rings in the polymer chain;
3. Heterocyclic polymers with a nitrogen atom: polypyrrole (PPy) and polypyridine (PPY);
4. Heterocyclic polymers with a sulfur atom: polythiophene (PTh) and polyethylene dioxythiophene (PEDOT), polyfuran (PFu), as well as polycyanamide (PCN) and polyvinylferrocene (pVFc).

**Figure 17:**
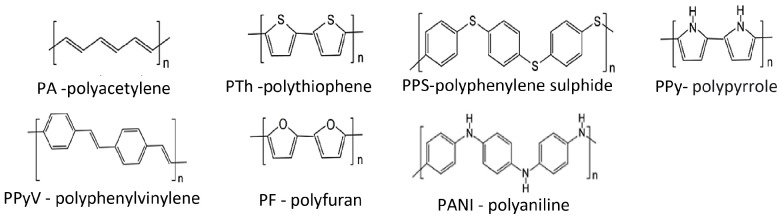
Structural formulas of conductive polymers.

Examples of structural formulas of conductive polymers are shown in Figure 17.

Conductive polymers, used as a sorption phase in the microextraction process, can interact with analytes through hydrophobic interactions, acid–base interactions, polar functional group interactions, ion exchange, hydrogen bonding, and π–π interactions. Because of their acid–base properties, CPs exhibit diverse extraction selectivity in relation to acidic and basic compounds. They also allow direct extraction of charged particles without derivatization or the application of complexing agents [50].

Examples of the use of conductive polymers in the TF-SPME technique, reported in scientific publications, are summarized in Table 4.

### 7.3. Molecularly Imprinted Polymers

**Figure 18:**
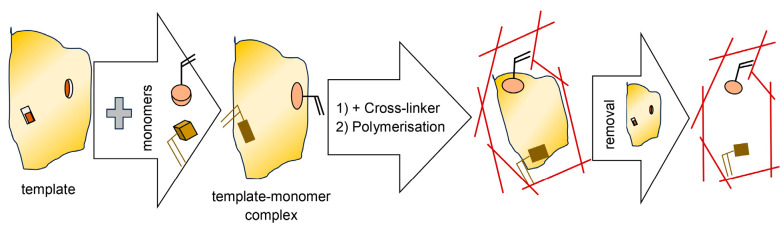
Synthesis diagram of MIPs [55].

Molecularly imprinted polymers (MIPs) are an essential group of the most selective sorption phases used in TF-SPME. MIP materials acquire their ability to selectively recognize an analyte during their synthesis, which proceeds with the participation of template molecules, functional monomers, a crosslinking reagent, an initiator, and a solvent. MIP synthesis occurs at high temperatures or by UV-light irradiation. During its course, template molecules combine with functional monomers to form complexes via covalent bonds or hydrogen bonds, π–π interactions, ionic interactions, and van der Waals forces. As a result of the polymerization reaction taking place around these complexes with the participation of a crosslinking agent, the complexes are immobilized in the polymer network. After synthesis, the template is removed, and the complex is degraded. Consequently, molecular cavities are formed with affinities for chemical structures identical or similar to those of the removed template. A scheme of the MIP synthesis is shown in Figure 18.

The template molecules used for the synthesis of MIP should exhibit chemical stability during the polymerization reaction and have functional groups that do not block the actual polymerization reaction and retain their capacity to interact with the functional monomer. A desirable feature of template molecules is also low molecular weight. Low-molecular-weight molecules are rigid and ensure the formation of permanent molecular cavities in the three-dimensional polymer network. Suitable functional monomers are selected for the template molecules, enabling the formation of chemical interactions leading to the formation of monomer–template transition complexes. Functional monomers also determine the formation of active sites in the MIP polymer that interact with analyte molecules.

The cross-linking reagent (cross-linker) forms bonds during MIP synthesis. The formed bounds stabilize the polymer structure, and thanks to them, the cross-linked structure remains stable after the removal of the template molecule. An important factor affecting the quality of the polymer product is the amount of cross-linking reagent used in the synthesis. Too little of it results in the polymer’s mechanical stability being lacking. At the same time, too much of it blocks functional groups and thus influences a reduction in the number of molecular cavities that are selective to the analyte [56].

In the synthesis of molecularly imprinted polymers, solvents of low polarity that generate the formation of large pores are used to provide the polymer material with the largest possible specific surface area. Solvent molecules of low polarity do not interfere with the interactions formed between the polymer and the template molecules, and simultaneously, they make it possible to obtain a material with high selectivity [57].

**Figure 19:**
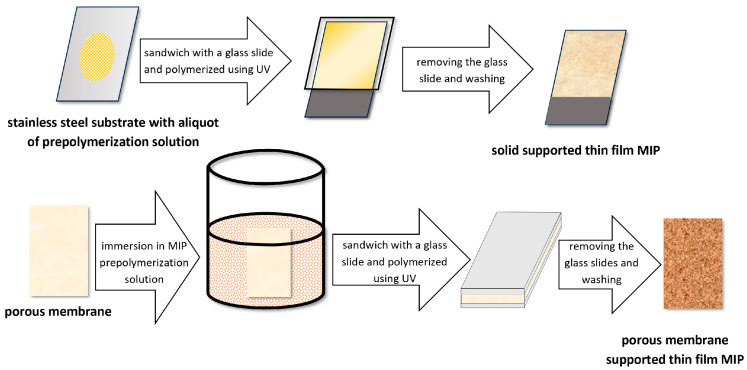
MIP-TFME preparation [58].

The MIP-TFME device is obtained by applying several microliters of the prepolymer solution to a silanized microscope slide or stainless substrate, then covering it with a coverslip and exposing it to UV light to initiate polymerization. The obtained polymer thin films are then washed with a suitable solvent to remove the template molecules from the polymer network. An alternative way of obtaining a MIP-TFME device involves immersing a porous membrane in a MIP prepolymer solution, placing the already-soaked membrane between two glass plates, and exposing it to UV light [58]. A diagram of the procedures described above is shown in Figure 19.

Examples of the application of MIPs in the TF-SPME technique, which have been reported in selected scientific publications, are summarized in Table 5.

### 7.4. Metal-Organic Frameworks

The term metal–organic frameworks (MOFs) is used to describe solid polymeric materials containing metal cations or secondary building units (SBUs) in their structure. SBUs are metallic clusters in which coordination bonds link the metal ion to multifunctional organic groups (linkers) such as carboxylates [65]. The most common transition metal ions used in MOF syntheses are, for example: Cr^3+^, Fe^3+^, Co^2+^, Zn^2+^, and Cu^2+^ acting as electron pair acceptors, which are provided by ligands containing atoms with free electron pairs, oxygen, nitrogen, or sulfur atoms. The function of ligands in MOF structures can be performed, inter alia, by polycarboxylic acids, for example, terephthalic, 1,3,5-benzenetricarboxylic [66].

MOFs are porous materials with the largest specific surface areas. MOF-5 is the first synthesized metal–organic skeleton-type structure, reported in “*Nature*” in 1999. Its skeleton is formed by zinc [Zn_4_O]^6+^ clusters and tetraphthalic acid anions. It has a specific surface area (Langmuir) of 2900 m^2^/g [67].

Considering vast specific surface areas, MOFs are characterized by good thermal and mechanical stability, homogeneous nanoscale cavity structure, uniform pore topology, ultra-low density, and high affinity to adsorption of various analytes [68]. The characteristics of MOF-type polymeric materials make them suitable for a variety of applications in science and technology (including catalysis, gas adsorption, and storage). Extraction techniques also apply to them as drug carriers and sorbents [69].

The adsorption of analytes on the surface of the MOF, used as a sorption phase, occurs through various types of interactions. Ligands in the crystal structure interact hydrophobically with analytes, while aromatic rings are present in linkers through π–π interactions. Ligands, containing oxygen and nitrogen polar functional groups, bind analytes through hydrogen bonds, ionic interactions, and dipole–dipole interactions. Metals at nodes of the crystal lattice, which have free coordination sites, enable acid–base Lewis interactions with molecules of the corresponding analyte [31].

Examples of the application of MOFs in the TF-SPME technique, described in scientific publications, are summarized in Table 6.

### 7.5. Carbon Nanomaterials

Carbon nanomaterials (CNMs) span a group of various allotropic carbon structures, which include, for example, fullerenes, carbon tubes, and graphene. CNMs are characterized by unique physical properties. They have a very high specific surface area, very high tensile, and high-temperature mechanical strength, as well as unique electrical and thermal conductivity. They also exhibit high affinity to many organic compounds. The properties enable extensive applications of carbon nanostructures in many fields of science and technology, including as adsorption materials [77].

#### 7.5.1. Graphene Materials

Graphene is an allotropic variety of carbon containing one or more layers of atoms in the sp^2^ hybridized state connected to each other on a single plane by strong σ bonds in a manner resembling a honeycomb structure.

The non-hybridized π atomic orbitals, perpendicular to the plane on which the carbon atoms make the graphene layer, overlap to form a delocalized, all-encompassing atomic plane π electron arrangement responsible for the specific optical, thermal, mechanical, and electrical properties of graphene [78]. The atomic structure of graphene can be considered basic to other allotropic carbon varieties such as graphite, carbon nanotubes, and fullerenes. Stacked graphene layers form the graphite structure. The rolled-up graphite layer constitutes a carbon nanotube, while curled up in a sphere constitutes fullerene [78].

Graphene and its derivatives, such as reduced graphene oxide (rGO) and graphene oxide (GO) are classified as graphene-based materials (GBMs). Graphene nanolayers allow the adsorption of molecules on both sides of the plane formed by carbon atoms. In contrast, in the case of nanotubes or fullerenes, adsorption of analyte molecules is not possible on the inner side of nanostructures due to steric crowding. Consequently, the specific surface areas of GBMs are very large (theoretical value for graphene: 2630 m^2^ g^−1^) and much larger than the specific surface areas of carbon nanotubes and fullerenes [79].

In regard to their high specific surface area, which is greater than that of SWCNTs and MWCNTs, rGO, and GO are used in extraction and microextraction techniques. Graphene-based materials adsorb various types of analytes via van der Waals-type interactions, electrostatic interactions, hydrogen bonds, π–π, dispersion forces, coordination bonds, and hydrophobic effect [80]. Due to π electron systems, rGO adsorbs aromatic rings of organic compounds via strong π–π interactions. The functional groups in graphene oxide play a key role in forming hydrogen bonds or electrostatic interactions with organic compounds containing functional groups with oxygen and nitrogen atoms in the molecules [81].

Table 7 summarizes examples of the application of graphene materials in the TFSPME technique presented in scientific publications.

#### 7.5.2. Carbon Nanotubes

Carbon nanotubes (CNTs) are an allotropic variety of carbon with cylindrical nanostructures that are made up of coiled monolayer sheets of sp^2^ carbon atoms linked together via covalent bonds. Depending on the number of layers of carbon atoms that make up the tube, CNTs are divided into single-wall carbon nanotubes (SWCNTs) and multi-wall carbon nanotubes (MWCNTs) consisting of several concentric graphene layers connected via van der Waals forces [90]. SWCNTs are nanostructures with diameters ranging from 1 nm to 10 nm, whereas MWCNT diameters are much larger, ranging from 5 nm to several hundred nanometers [91].

Carbon nanotubes are characterized by high thermal, mechanical, and chemical stability, a specific surface area of between 150 and 1500 m^2^ g^−1^, as well as high sorption capacity. The large sorption capacity of CNTs is a consequence of their large length-to-width ratio. Unlike activated carbons, carbon nanotubes are not porous materials. Adsorption in porous materials is related to the diffusion of analyte particles deep into the pores of the sorbent, whereas sorption-active sites are located on the surface of CNTs and in the spaces between the rolled graphene layers that form MWCNTs.

Nanotubes are easily accessible to analyte particles during adsorption. Compared to desorption occurring in the pores of a porous sorbent, the nanotubes enable faster thermal desorption of analytes and solvents. Due to the mechanism of adsorption and desorption, which is not controlled by diffusion, carbon nanotubes are used to concentrate a wide range of analytes [92].

The hexagonal carbon backbone of graphene layers is responsible for the low affinity of CNTs to polar molecules; as a result, pure carbon nanotubes are used as a sorption material in the microextraction of hydrophobic analytes. Carbon nanotubes, functionalized by oxidation reaction, are used in the microextraction of polar analytes. The redox reaction allows the connection between carbon atoms and -OH, -COOH, and -NH_2_ groups, among others.

Sorption of analytes on pure and functionalized CNTs can occur via hydrogen bonds, π–π interactions, electrostatic forces, and hydrophobic interactions. π–π interactions between graphene layers of CNTs and molecules of analytes containing benzene rings in their structure, enable extraction using carbon nanotubes as a sorption phase of polycyclic aromatic hydrocarbons (PAHs), pharmaceuticals, phthalates, pesticides, and parabens. The extraction of metal ions, positively charged particles, and polar molecules occurs via hydrogen bonds, and electrostatic interactions between them and the functional groups present on the CNT surface [31].

Table 8 summarizes examples of the application of carbon nanotubes in the TF-SPME technique, which are used to analyze environmental and food samples and have been described in scientific publications.

### 7.6. Silica Materials

**Figure 20:**
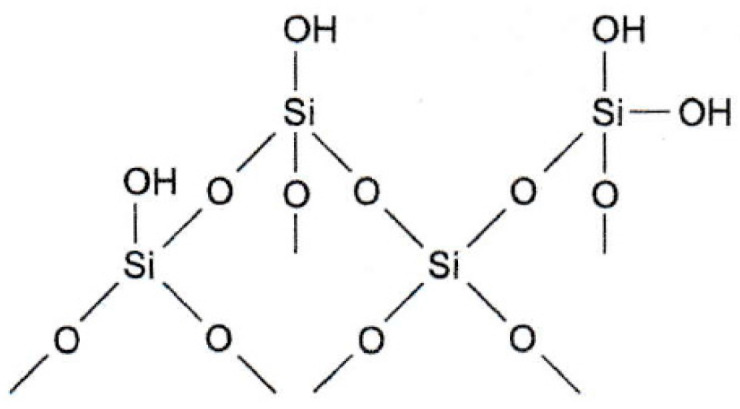
Chemical surface structure of silica gel.

Silica gel SiO_2_ (silica) and chemically modified silica are materials commonly used in analytical chemistry, both in extraction techniques, filtration, and as a stationary phase in chromatography. Hydrated silicon oxide with the general formula SiO_2_-*n*H_2_O (hydrated silica) is an amorphous and porous substance whose spatial network is formed by tetrahedral SiO_4_. On the surface of its microparticles, there are silanol (≡Si-OH) or siloxane (≡Si-O-Si≡) groups (Figure 20).

The active groups of silica can react with organic compounds to form so-called bound phases. The bonded phases can contain bonds of the following types:

1. Ester, formed in the reaction of a silanol group with an alcohol:
  ≡Si-OH + R-OH→≡Si-O-R + H_2_O
2. Carbon, formed in the reaction of a silanol group with thionyl chloride and then with an organometallic compound:
  ≡Si-OH + SOCl_2_→≡Si-Cl+ SO_2_+HCl
  ≡Si-Cl + R-Li→≡Si-R+ SO_2_+LiCl
3. Siloxane, formed by the reaction of the silanol group with organ chlorosilanes:
  ≡Si-OH + Cl-SiR_3_→≡Si-O-SiR_3_ + HCl.

The phases, formed by the reaction of silanol groups of silica with organ chlorosilanes, are characterized by the non-polar (hydrophobic) nature of the surface, as there are alkyl chains bound to silicon atoms. The polarity of the surface of silica, modified with siloxane bonds, depends on the number of bound alkyl chains and their length, as well as the number of unbound -OH groups. It decreases with an increase in the length of the carbon chains and with a decrease in the number of free hydroxyl groups. Of most significant practical importance are the bonded phases in which alkyl groups, with two (C2), eight (C8), or eighteen (C18) carbon atoms, are attached to the surface of the silica gel. A non-polar octadecyl phase with eighteen carbons in the chain (C18) is the most commonly used in extraction and microextraction techniques. It is applied in the microextraction of non-polar analytes, such as polycyclic aromatic hydrocarbons.

Table 9 summarizes examples of the application of C18 silica in the TF-SPME technique used to analyze environmental, biological, and food samples, which have been described in scientific publications.

### 7.7. Aptamers

Aptamers are single-stranded oligonucleotides of ribonucleic acid (RNA) or deoxyribonucleic acid (DNA), most often between 20 and 80 nucleotides in length. They form three-dimensional undulating conformations and, like antibodies, have the ability to bind to a variety of biological and molecular targets. The main characteristics of aptamers are their high specificity and affinity to binding well-defined biomolecules of different sizes such as nucleotides, amino acids, biopolymers, polysaccharides, peptides, or proteins.

Aptamers can combine with molecules of varying chemical structure, for example, metal ions, enzymes, regulatory proteins, growth factors, antibodies (both mono- and polyclonal), lectins, vitamins, antibiotics, amino acids, peptides, nucleotides, or organic dyes. Their characteristics enable them to be used as highly selective sorbents in microextraction to the solid phase. Used as selective microextraction sportive phase, aptamers must be bound to solid supports. The aptamer-binding substrate should be characterized by chemical and biochemical inertness and good mechanical stability. The aptamer immobilization procedure must preserve its affinity to the target analyte [105].

Table 10 summarizes examples of the use of aptamers in the TF-SPME technique, which are used to analyze environmental, biological, and food samples and have been described in scientific publications.

### 7.8. Ionic Liquids and Poly(Ionic Liquids)

**Figure 21:**
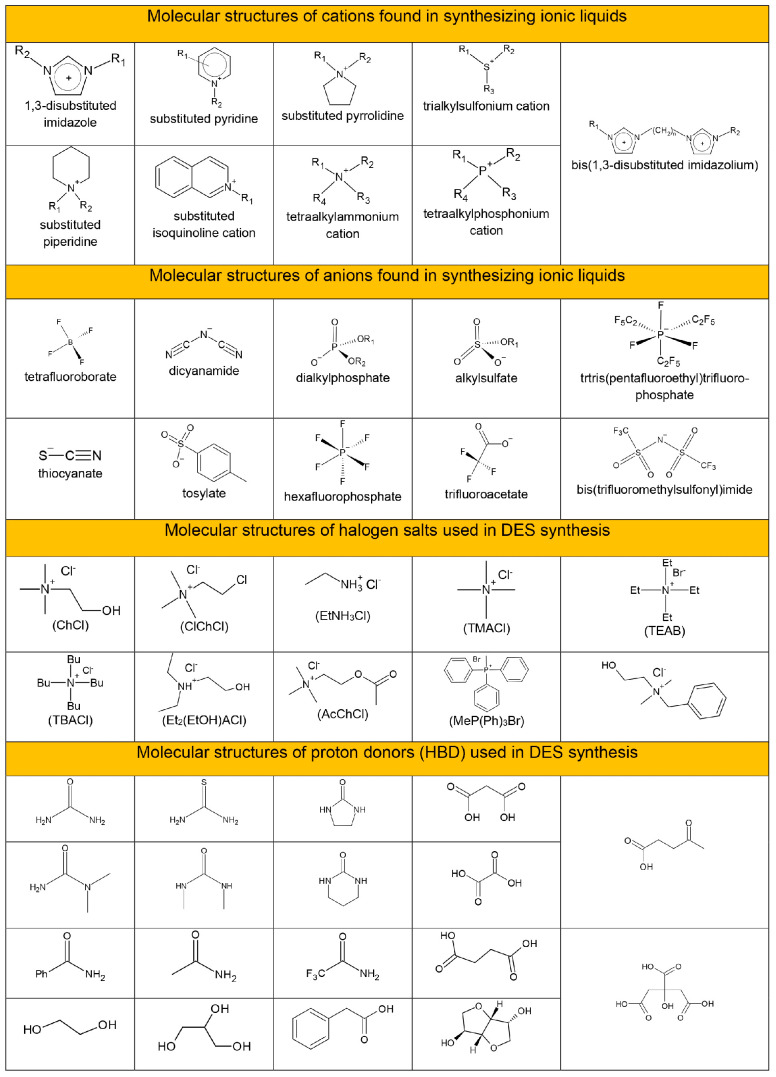
Molecular structures of substrates found in synthesizing ILs and DES [110,111].

Ionic liquids (ILs) are salts containing organic cations and organic, or inorganic anions with a melting point equal to or below 100 °C. The cation of an ionic liquid is usually a branched organic structure with low symmetry. The cations forming ionic liquids include imidazolium (IM), pyrrolidine (Pyrr), pyridine, tetraalkylammonium, or tetralkylphosphonium. The anions included in the ionic liquids are the halide anions Cl^−^, Br^−^ I^−^, but also triflate ([TfO^−^]), bis(trifluoromethylsulfonyl) imide ([TFSI^−^]), tetrafluoroborate ([BF_4_^−^]), or hexafluorophosphate ([PF_6_^−^]) [109]. Figure 21 show the molecular structures and nomenclature of various cations and anions commonly found in synthesizing second- and third-generation ionic liquids [110].

Ionic liquids are among chemical compounds with unique physicochemical properties, such as high thermal stability, near-zero vapor pressure, high viscosity, non-flammability, and the ability to dissolve a wide range of organic and inorganic chemicals. Physical properties such as miscibility and thermal stability depend primarily on the anion. In contrast, other properties, such as viscosity and surface tension, are controlled by the length of the alkyl chain on the cation and the shape and symmetry of the cation [110]. The negligible vapor pressure of ionic liquids, their good thermal stability, and controllable viscosity, as well as their miscibility with water and organic solvents, are the main properties that make ionic liquids applicable in microextraction to the solid phase. The high viscosity of ILs facilitates their application to the substrate and positively influences the homogeneity of the sorption film. Simultaneously, their liquid state makes it easier to conduct the extraction process due to the higher diffusion rates of analytes compared to solid phases. The ability to design ILs by matching the type of cation and anion to the target analyte is a factor that determines high selectivity and sensitivity to determinations carried out using solid-phase microextraction [112]. Ionic liquids (ILs) are used in SPME microextraction, while poly(ionic liquids) PILs—in TF-SPME techniques. Poly(ionic liquids) are a subgroup of polyelectrolytes that can be obtained by polymerizing ionic liquid monomers or post-polymerization processes [109]. The polymer chains of PILs repeat ionic units: cationic, anionic, or zwitterionic. In the cationic structure, groupings endowed with a positive electrical charge are covalently attached to the polymer backbone. Typical cationic PILs are based on imidazole, pyridine, pyrrolidine, and quaternary ammonium cations. Groupings with a negative electrical charge, such as sulfonates, carboxylates, and phosphates, covalently link to the polymer chain in anionic PILs. In the case of zwitterionic PILs, both cations and anions are covalently attached to the polymer chains [113].

The physicochemical characteristics of polymeric ionic liquids, such as high thermal, chemical, and mechanical stability and excellent plasticity, make them applicable in various fields of science and technology, including microextraction techniques. ILs, used as sorption phases in microextraction, provide high extraction efficiencies for a wide range of compounds due to their porous structure and the presence of functional groups in the polymer chain that enable a variety of interactions with the analyte molecules [113].

Table 11 summarizes examples of the use of polymeric ionic liquids in the TF-SPME technique, which are used to analyze environmental, biological, and food samples and have been described in scientific publications.

### 7.9. Deep Eutectic Solvents

Deep eutectic solvents (DES) are systems formed from a mixture of various types of cationic and anionic Lewis or Brønsted acids and bases [118]. The ions in the mixture, as a result of hydrogen bonding, form a eutectic mixture. A eutectic mixture is characterized by a much lower melting point than the melting point of each of its components [111]. They are mainly obtained by mixing the quaternary ammonium salt with metal salts or a hydrogen bond proton donor (HBD). A hydrogen bond proton donor (HBD) has the ability to form a complex with the halide anion as a hydrogen bond acceptor (HBA) from the quaternary ammonium salt. Figure 21 shows quaternary ammonium salts and hydrogen bond proton donors (HBD) widely used in DES formation [111].

DES have similar properties to ionic liquids, but unlike them, they are less toxic, non-flammable, and biodegradable [119]. The physicochemical properties of deeply eutectic solvents depend both on the properties of each component of the eutectic mixture, and on the interaction between them. The values of the physical quantities, such as freezing point, viscosity, electrical conductivity, and pH value, can be modified by an appropriate combination of different quaternary ammonium salts (e.g., ChCl) and hydrogen bond donors (HBD) [111]. The density of deeply eutectic solvents ranges from 1.041 to 1.63 g∙cm^−3^. Its value is influenced by temperature (with an increase in temperature, the density of DES decreases linearly) and the molar ratio of the components of the eutectic mixture [120]. Most deep eutectic solvents have higher viscosity and surface tension at room temperature than conventional solvents. The extensive interconnection of DES-forming particles via hydrogen bonds, van der Waals forces, and electrostatic interactions are responsible for the high viscosity and surface tension values [120].

DES are increasingly used in analytical chemistry, mainly as mobile phase modifiers in chromatography and extractants in various microextraction techniques to the liquid phase, and in recent years, also in microextractions to the solid phase [121]. A significant number of DES between room temperature and 70 °C occur in the liquid state [111]. However, there are solid eutectic systems at room temperature that can be used as sorption phases in microextraction to the solid phase.

Table 12 summarizes examples of the use of DES in the TF-SPME technique, which has been described in scientific publications, to perform surface water analysis.

## 8. TF-SPME with Thermal and Solvent Desorption

Desorption of analytes from the TF-SPME device can be carried out at high temperatures or in an organic solvent. Thermal desorption requires the sorptive phase and its support to be highly thermally stable. Due to the larger size of the TF-SPME device than the SPME fiber, performing thermal desorption of analytes from the TF-SPME sorbent involves the use of a gas chromatograph column dispenser adapter. The adapter, a thermal desorption unit (TDU), is an accessory for the GC gas chromatograph. During thermal desorption, the sorptive phase is heated with hot gas to release all volatile analytes adsorbed on it. The increase in temperature of the sorption phase increases the partition coefficient of the analytes and consequently promotes their release from the sorbent solid phase into the gas phase. Thermal desorption in the TDU of the gas chromatograph allows direct introduction of all analytes, except non-volatile substances, into the chromatographic column. The non-volatile substances remain on the TF-SPME device [9].

Liquid desorption is commonly used in conjunction with liquid chromatography. It uses a small amount of organic solvent (or a mixture of water and multiple organic solvents) to re-extract all compounds from the solid phase of the TF-SPME device to the liquid phase. After desorption, the analytically enriched organic solvent is injected into the liquid chromatograph. Desorption in the solvent is most commonly used while analyzing non-volatile, thermally labile compounds and biomolecules. Diffusion of analytes in the liquid occurs more slowly than in the gas phase, and consequently, desorption in organic solvents is a longer process than thermal desorption [8].

## 9. Application of the TFME Technique in the Analysis of Organic Compounds

The TF-SPME technique has been used to concentrate and analyze a wide range of organic compounds in aqueous, biological, and food matrices for over twenty years. In addition to the possibility of preparing samples in the laboratory, the TF-SPME technique allows sampling onsite [43] and in vivo [37,123,124]. The TF-SPME technique has been successfully used to extract in vivo prohibited substances and oral cancer markers from human saliva [125] and analytes from human skin [37].

**Figure 22:**
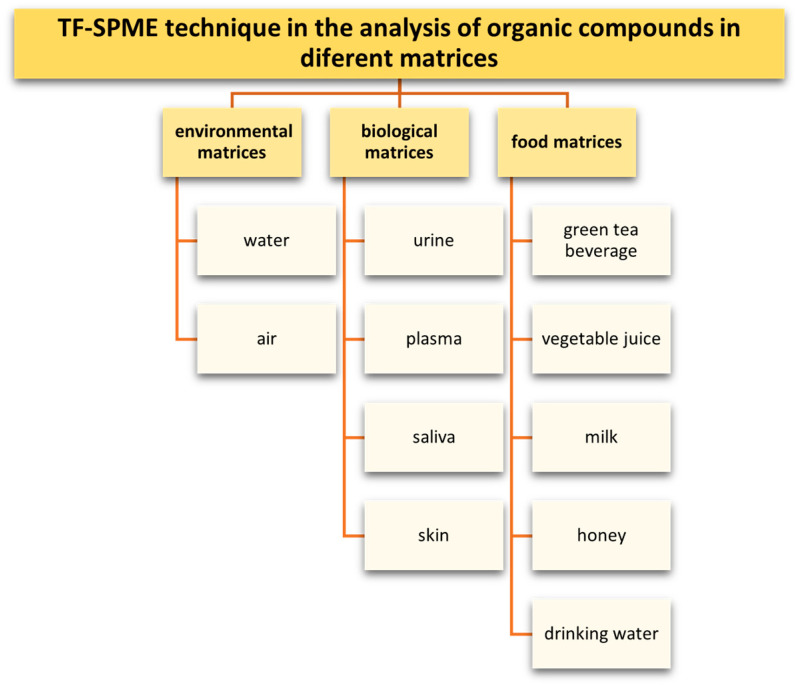
Matrix types containing analytes determined using the TF-SPME technique.

The different types of matrices containing analytes, whose determination by TF-SPME have been described in scientific publications, are shown in Figure 22.

Organic analytes whose determination procedures were developed using TF-SPME mainly belong to the following:

1. Compounds that are environmental contaminants in aqueous and food matrices (polycyclic aromatic hydrocarbons, polycyclic aromatic sulfur heterocycles (PASHs), plant protection products, phenols, chlorophenols, alkylphenols, bisphenol A);
2. Preservatives determined in aqueous matrices (parabens and personal care products PCPs);
3. Biologically active compounds determined in aqueous and biologic matrices (drugs from various therapeutic groups including nonsteroidal anti-inflammatory drugs, antibiotics, antidepressants, tranquilizers, opioids, anti-cancer drugs, sex hormones, doping agents, narcotics).

Polycyclic aromatic hydrocarbons (PAHs) and polycyclic aromatic sulfur heterocycles PASHs, along with polychlorinated biphenyls (PCBs), organochlorine, and organophosphate pesticides are among the hazardous pollutants of surface water and food. Due to the toxicity of PAHs, environmental pollution by polycyclic aromatic hydrocarbons is a factor that has a very negative impact on the human and animal bodies. They negatively affect endocrine, reproductive, and developmental processes [126]. For many compounds, among several hundreds of known PAHs, the initiation of tumor transformations has been proven. Similarly to PAHs, polycyclic aromatic sulfur heterocycles PASHs, such as dibenzothiophene (DBT), are components of petroleum products with mutagenic and carcinogenic effects.

Analytical procedures have been developed for the determination of PAHs in aqueous matrices, using TF-SPME with sorption phases: PDMS [34] and MWCNTs/CTA [100], while in food matrices, using MWCNTs/Agr [95]. In the determination of PASHs in water samples, MIP [59] was used as the sorption phase (Table 13 and Table 14).

The need to maintain global food production at a level sufficient to feed the earth’s growing population necessitates the use of crop protection products. These products, while increasing yields in agricultural production, also contribute to the pollution of the environment with hazardous organic compounds. This underscores the critical need to monitor even trace amounts of pesticides and fungicides in treated water for drinking and food products [127].

Analytical procedures have been developed for the determination of pesticides and fungicides in aqueous matrices using TF-SPME with sorptive phases in the form of PDMS/DVB [42], polypropylene/polyamide nanofibers [52], MOFs [74], MWCNTs/CTA [96], (MOF)/(PU) [128], MIP [54], and DES/CTA [127]. For the determination of plant protection agents in food samples, sorption phases were used in TF-SPME devices such as HLB/PTFE AF [129], POM@UIO-66-NH2/GO [130], POM@UIO-66-NH2/GO [131], UiO-66/PS [132], and PU/PMMA [133] (Table 13 and Table 14).

Phenol and its derivatives, chlorophenols, alkylphenols, and bisphenol A, are another group of organic compounds, along with PAHs and pesticides, that pollute surface waters and pose a threat to aquatic organisms and human health. They come from a variety of sources, such as oil extraction and processing, wood and coal pyrolysis, industrial organic synthesis, and plastic production [134]. Chlorophenols are persistent toxic substances that cause histopathological changes and mutations in aquatic organisms, and some are probable human carcinogens, such as pentachlorophenol (PCP). Alkylphenols are endocrine disruptors and have widespread effects on the health and function of a range of aquatic organisms, as well as humans [135]. Bisphenol A, as a polymer additive, is used to manufacture polycarbonate plastic bottles used to store beverages and infant milk. BPA is released from the polycarbonate bottle into the liquid it contains. As a result, an infant’s consumption of liquids from polycarbonate bottles contributes to an increase in the concentration of BPA in urine. BPA exhibits xenobiotic activity, estrogenic activity, and endocrine-disrupting properties. In addition, bisphenol A has been linked to decreased fertility and cancer. The adverse effects of bisphenol A on human health imply the need for its determination in drinking water and food matrices [136].

The authors of the referenced TF-SPME studies used sorptive phases to determine phenolic compounds in aqueous matrices: MIP [61,63], MWCNTs-COOH/PDMS [97], MWCNTs-COOH-Ch/PP [99], PVA/PVP/PES [136], and polyamide-coated paper [137]. In the determination of chlorophenols in the food matrix, AC/GO [138] was used as the sorption phase (Table 13 and Table 14).

Parabens are a family of synthetic p-hydroxybenzoic acid esters that includes methylparaben (MP), ethylparaben (EP), propylparaben (PP), butylparaben (BP), isobutylparaben (IBP), isopropylparaben (IPP), benzylparaben (BeP), and heptylparaben (HP) and their respective sodium salts. They are used as preservatives in everyday products such as cosmetics, food, toiletries, etc. [139]. European Union legislation has restricted the use of parabens in cosmetics because they can cause allergic reactions and disrupt human hormonal metabolism [140]. However, limits have yet to be set for the concentrations of parabens in environmental samples [121].

The researchers used TF-SPME to determine parabens in aqueous matrices using the following sorption phases: DES [119], PDMS/DES [121], and CA-MIL-101(Cr)@CNFs [141] (Table 13).

Non-steroidal anti-inflammatory drugs (NSAIDs) are among the most widely used pharmaceuticals, often applied to suppress inflammation, treat allergies, and reduce pain. Due to their hydrophilicity and stability in water, NSAIDs can persist in the aqueous phase for long periods and thus adversely affect aquatic organisms and human health [142]. Side effects of non-steroidal anti-inflammatory drugs include cardiovascular disorders, gastrointestinal disorders, increased blood pressure, and kidney damage. Thus, monitoring the level of a non-steroidal anti-inflammatory drug in a patient’s body can significantly contribute to better diagnosis and treatment of effects caused by, among other things, its overdose [143].

In the determination of non-steroidal anti-inflammatory drugs in aqueous matrices, the authors of the publications used TF-SPME, in which the following sorption phases were applied: MWCNT/Agr-Ch [94], while in biological matrices: CY-GO-LDH [82], p-PIL-AcO [115], MOF-5 [143], and LDH/GO/PVDF [84] (Table 13 and Table 15).

Glucocorticoids are among the drugs used to treat allergies, asthma, and estrogen and autoimmune disorders [144]. They enter the aquatic environment as a result of wastewater discharges directly into the environment or as an effect of incomplete removal from contaminated water in wastewater treatment plants [145]. The presence of glucocorticoids in concentrations at ng L^−1^ levels is a factor that adversely affects aquatic organisms [146]. In the determination of glucocorticoids in aqueous matrices, the authors of the publications used TF-SPME, in which they used SDS-MWCNTs/PP [93] as a sorption phase (Table 13).

Antibiotics are widely used in both human and veterinary medicine. Global consumption of antibiotics in industrial animal husbandry is increasing year by year. Through animal manure, which is used as fertilizer in agricultural production, antibiotics enter the soil in small amounts and then into post-surface water. Ineffective degradation of pharmaceuticals during wastewater treatment can lead to contamination of surface water, groundwater, and, consequently, drinking water. Residues of antibiotics in water can enter the food chain and adversely affect human health, including causing allergic reactions. As a consequence, there is a need to determine the level of antibiotic concentrations in environmental waters [147]. In the determination of antibiotics in aqueous matrices, the researchers used TF-SPME, in which they used sorption phases: p-Poly-(MMA-IL)FP [147] and PVA-SA-βCD [148] (Table 13).

Steroid hormones are a group of hormones that have a steroid skeleton in their chemical structure. Natural steroid hormones are usually synthesized from cholesterol in the adrenal cortex. Steroid hormones regulate such physiological processes as, for example, carbohydrate, protein, and fat metabolism (glucocorticoids) and water and mineral balance (mineralocorticoids). Estrogens, including estradiol, are involved in the development of primary and secondary female sexual characteristics, while progestins (e.g., progesterone) are involved in the maintenance of pregnancy. Androgens (e.g., testosterone) control the development and maintenance of reproductive function and are responsible for secondary sexual characteristics in men [149]. Estrogens play an important role at different stages of human development. In the case of post-menopausal women, estrogen levels are low, and abnormal levels of estrogen concentrations may be associated with the development of breast cancer [150]. In the microextraction of hormones by the TF-SPME technique from biological matrices, researchers have used sorption phases: PANI [53,151], C18/PAN, PS/DVB [149], MIL-53(Al)/PVDF, MIL-53(Fe)/PVDF, MIL-100(Fe)/PVDF, MIL-101(Cr)/PVDF, and UiO-66(Zr)/PVDF [72] (Table 15). From the aqueous matrix, estrogens were extracted into the C18/PAN sorption phase [47] (Table 13).

One of the most prevalent mental illnesses today is depression. Antidepressants impede serotonin reuptake in presynaptic neurotransmission. Monitoring drug concentrations in the patient’s blood or urine is essential to determine the optimum therapeutic dose for the patient, to increase the efficacy of therapy, and to reduce the risk of laccase intoxication [62]. Recent publications have presented analytical procedures for the determination of sedative and antidepressant drugs in biological matrices using TF-SPME with sorption phases: C18/glue [25], C18-TEOS [101], C18/PAN [44], GO/CS [152], Ni-Co MOFs-PAN [153], MIP [62], and poly(vinyl alcohol) (PVA)/citric acid(CA)/β-cyclodextrin/Bi2S3@g-C3N4 [154] (Table 15).

Opioids include all substances that act on opioid receptors: natural opiates, semi-synthetic poppy alkaloids, their synthetic derivatives, and endogenous peptides. The main use of opioids is to manage severe pain (post-operative, post-traumatic, cancer), often chronic. Using them for purposes other than therapeutic can be dangerous and addictive [155]. Procedures for the labeling of opioids, for example, codeine, tramadol, and fentanyl in biological matrices, have been developed using TF-SPME microulceration with sorption phases in the form of aptamer/cellulose [106,108], Ni(DMG)2-NiO-Cell [156], and octyl-cyanopropyl/PAN [157] (Table 15)

Beta-blockers are essential clinical drugs that are used to treat cardiovascular diseases such as hypertension, irregular heart rhythms, strokes, and angina. Additionally, β-blockers improve cardiac function by relaxing muscles and reducing heart rate, which is often abused by athletes competing in professional sports [87]. Procedures for the determination of beta-blockers in biological matrices have been developed using TF-SPME with sorption phases such as GO/PEG [87] and g-C3N4/N6 NC [158] (Table 15).

Table 13, Table 14 and Table 15 provide details of the quantification of analytes in aqueous, food, and biological matrices using TF-SPME reported by researchers in the last few years.

**Table 13:** Applicability of TF-SPME technique for quantitative analysis of different analytes in water matrix.

Analyte	Sorption Phase	Detection	LOD	Recovery [%]	RSD [%]	Ref.
PAHs	PDMS	GC-MS	-	-	6–11	[34]
	MWCNTs/CTA	HPLC-UV	0.02–0.09 ng/mL	99–101	1–8	[100]
Polycyclic aromatic sulfur heterocycles	MIP	GC-MS	0.029–0.166 µg L^−1^	-	≤6.0	[59]
Pesticides/fungicides	DVB/PDMS	GC-MS	0.01–0.25 μg L^−1^	90–130	2–20	[42]
		GC-MS	1.0–4.0 ng L^−1^	71–124	3–20	[43]
Pesticides/fungicides	PPy/PA	GC-MS	50 ng L^−1^	96–98	4	[52]
	amino-Zr-MOF/PAN	CD-IMS	0.6 μg L^−1^	-	11	[74]
	MWCNTs/CTA	GC-MS	1 μg L^−1^	-	≤20	[96]
	MIP	LC-MS/MS	0.002–0.02 μg L^−1^	90–110	<15	[60]
	PAMAM@GO-PVDF	HPLC-UV	0.12–0.20 μg L^−1^	98–99	-	[89]
	MOF/PU	GC-MS	0.005–0.1 μg L^−1^	72–110	4–5	[128]
	DES/CTA	GC-MS	0.4–1.3 μg L^−1^	69–103	3–14	[127]
	PVA/CA/C)/AV	HPLC-UV	-	86–97	6–7	[159]
	DES	HPLC-UV	-	72–94	3–11	[160]
	DVB/PDMS	GC-TMS	-	-	-	[40]
VOCs/SVOCs		GC-MS	-	-	-	[24]
N-nitrosamines	DVB/PDMS	GC-MS	3 ng L^−1^	-	8	[22]
Chlorobenzenes	PANI-N6	GC-MS	19–33 ng L^−1^	93–103	5–14	[54]
Phenols	MIP	UHPLC-PDA	0.1–2 μg L^−1^	85–100	1–14	[61]
		LC-MS	-	-	-	[63]
	MWCNTs-COOH/PDMS	HPLC-UV	1–2 μg L^−1^	64–90	-	[97]
Polychlorinated biphenyls	MWCNTs-COOH-Ch/PP	GC-MS	<0.60 ng L^−1^	86–104	0.17–5.01	[99]
Bisphenol A	PVA/PVP/PES	FS	0.3 ng mL^−1^	84–96	5–10	[136]
Phthalates, alkylphenols, bisphenols	Polyamide-coated paper	HPLC-DAD	1.5–7.6 μg L^−1^	-	≤24%	[137]
Sulfonamides	p-Poly-(MMA-IL)	HPLC-DAD	0.14–0.52 µg L^−1^	90–110	10	[116]
Aniline	poly-(MMA-BVImBr)	LC-MS/MS	0.5 μg L^−1^	91–96	8.3	[117]
Parabens	DES	HPLC-UV	0.018–0.055 ng L^−1^	68–94	4–7	[119]
Parabens	PDMS/DES	HPLC-UV	0.023–0.062 ng mL^−1^	79–88	3–6	[121]
	CA-MIL-101(Cr)@CNFs	HPLC-DAD	11 ng L^−1^	92–100	<5	[141]
Formaldehyde	DES	HPLC-UV-Vis	0.15 ng mL^−1^	78–99	3–5	[122]
Personal care products	MIL-100(Fe)/PS/cellulose	HPLC-PDA	7.5 µg·L^−1^	78–128	11	[75]
Flame retardants:	ZIF-8@N-rGO	HPLC	0.03–0.14 ng L^−1^	89–106	-	[161]
Nonsteroidal anti-inflammatory drugs	MWCNT/Agr-Ch	HPLC-UV	0.89–8.05 ng mL^−1^	-	<5	[94]
Estrogens	C18/PAN	LC-UV	1.2–1.6 ng mL^−1^	87–109	3–6	[47]
Glucocorticoids	SDS-MWCNTs/PP	UHPLC-MS	0.019–0.098 ng mL^−1^	-	2–4	[93]
Carbamazepine	C18/SCX	DESI-MS	<ng L^−1^	-	-	[104]
Triclosan	C18/SCX	DESI-MS	<ng L^−1^	-	-	[104]
Antibiotics: sulfonamides, tetracyclines, fluoroquinolones, penicillin, macrolides	p-Poly-(MMA-IL)FP	LC-MS/MS	0.05–4.52 μgL^−1^	79–127	1–12	[147]
Antibiotics: Amoxicillin, enrofloxacin, tetracycline, doxycycline	PVA-SA-βCD	HPLC-UV	0.02–0.03 μgL^−1^	70–100	1–2	[148]
Illicit drugs: methamphetamine ketamine methaqualone	DVB/PDMS	GC-MS	For methamphetamine: 5.5 ng L^−1^ For ketamine: 2.0 ng L^−1^ For methaqualone: 1.1 ng L^−1^	95–111	<6	[162]
Chlorpyrifos, triclosan, tonalide	CTA with plasticizers	GC-MS	0.05–0.42 μgL^−1^	>80	<10	[163]
Haloacetic acids	PDMS HLB/PDMS Carboxen/PDMS	GC-ECD	-	51–92	7–19	[164]
Sexual hormones: 17β-estradiol, 17α-ethinylestradiol, estrone, progesterone, medroxyprogesterone acetate, hydroxyprogesterone	CTA/NPOE CTA/DBS	HPLC–MS/MS	0.1–5.7 ng L^−1^	>60	<12	[165]
Organic pollutants: benzene, 2-hexanone, hexanal, α-pinene, limonene, eucalyptol, 2-nona- none, 2-nonanol, 2-undecanone, ethyl nonanoate, 1-undecanol, ethyl undecanoate	GO/PS-DVB s	GC-MS	0.4–8.1 ng L^−1^	78–111	2–6	[166]

**Table 14:** Applicability of TF-SPME technique for quantitative analysis of different analytes in food matrices.

Matrix	Analyte	Sorption Phase	Detection	LOD	Recovery [%]	RSD [%]	Ref.
Green tea beverage	PAHs	MWCNTs/Agr	HPLC-UV	0.1–50 ng L^−1^	91–107	-	[95]
Vegetable juice	Codeine, acetamiprid	Aptamer/cellulose	ESI-IMS	1.8–3.7 ng mL^−1^	-	2–6	[106]
Honey, pork, chicken, milk	Sulfonamides: sulfathiazole, sulfamerazine, sulfadimidine, sulfamethoxazole, sulfisoxazole	MIL-101(Cr)/CC	HPLC-PDA	2.5–4.5 μg mL^−1^	82–114	<9	[167]
Milk	Sulfathiazole	HVImBr/MMA	SF	0.07–0.23	84–107		[114]
	Phthalates	C18-FMSNs/PAN	HPLC	0.096–0.26 ng mL^−1^	86–110	<7	[168]
Honey	Macrolides, lincosamides	ZIF-8@GO	UPLC-MS/MS	0.1–04 μg kg^−1^	68–107		[88]
Honey, tea	Chlorophenols	ACGO	HPLC-UV	0.03–0.13 μg mL^−1^	-	3–6	[138]
Drinking water	Anti-inflammatory antibacterial drugs	DVB/PAN	LC-ESI-MS/MS	ng L^−1^	-		[41]
Cod liver oil	Polychlorinated n-alkanes	HLB/PDMS	GC-MS	0.07–0.22 μg/g	-	2–12	[169]
Apple, tomato	Benzoylurea insecticides	PAN/ZIF8@E coli	HPLC-UV	0.12–0.15 μgL^−1^	93–110	≤8	[170]
Fruit juice, black tea	Flavonoids: morin, quercetin	Co_3_O_4_@GO-Nylon-6	HPLC-UV	For morin: 1.3 μgL^−1^ For quercetin: 1.6 μgL^−1^	For morin: 73 For quercetin: 64	<5	[171]
Fruit and tea beverages	Pesticides	polyurethane	GC-ECD	0.001–0.015 μgL^−1^	77–106	-	[172]
Apple juice		HLB/PTFE AF	GC-MS	1.0–5.0 ng mL^−1^	-	≤20	[129]
Cereal		PVA/MCS/HC-POF	HPLC-UV	≤4.0 ng mL^−1^	63–79	≤7	[130]
Vegetables and fruits		POM@UIO-66-NH_2_/GO	HPLC-UV	0.31–0.34 μgL^−1^	89–102	2–4	[131]
Vegetables and fruits		UiO-66/PS	GC	1.5–3 μg kg^−1^	88–96	5–7	[132]
Carrot juice, apple juice, strawberry juice		phosphotungstic acid/polyvinylidene fluoride membrane.	HPLC-UV-Vis	0.29–0.31 μgL^−1^	96–105	4–6	[173]
Fruit juice, tea	Neonicotinoid insecticides	PU/PMMA	UPLC-MS/MS	0.001–0.1 μgL^−1^	81–108	-	[133]
Fruit juice	Thiram fungicide	Silver nano network/silicon wafer	SERS	0.01 μgL^−1^	-	7	[174]
Milk, honey, fruits, vegetables	Conazole fungicides	MIL-88A@CNTs	CD-IMS	For penconazole: 0.30 ng mL^−1^ For propiconazole: 0.50 ng mL^−1^	86–97	5–7	[175]
Dry chili, chili powder, dry Sichuan pepper, Sichuan pepper powder	Rhodamine B	COF-117-PTFE	HPLC-FLD	0.007 μgL^−1^	68–71	7	[176]

**Table 15:** Applicability of TF-SPME technique for quantitative analysis of different analytes in biological matrices.

Matrix	Analyte	Sorption Phase	Detection	LOD	Recovery [%]	RSD [%]	Ref.
Urine	Doping agents	C18/PAN	LC-MS	0.25–10 ng mL^−1^ LOQ	85–130	<20	[45,46]
	Hormones	PANI	HPLC-FLD	0.30–3.03 μg L^−1^	71–115	≤12	[53]
	Steroidal hormones	C18/PAN PS/DVB	UHPLC-ESI-QTOF/MS	-	74–99	-	[149]
	Estrogens	MIL-53(Al)/PVDF MIL-53(Fe) /PVDF MIL-100(Fe) /PVDF MIL-101(Cr) /PVDF UiO-66(Zr) /PVDF	HPLC-FLD	0.005–1 ng mL^−1^	80–103	≤11.4	[72]
	Urinary androgens	-	HPLC-QqQ/MS	0.04–0.09 ng mL^−1^	-	-	[177]
	Caffeine	ZIF-8/LDH/GO/PVDF	HPLC-UV		-	-	[73]
	Aldehydes	MOF-199/PS	HPLC- VWD	4.2–17.3 nmol L^−1^	82–112	2–13	[76]
	Non-steroidal anti-inflammatory drugs	CY-GO-LDH	HPLC-UV	0.25 μg L^−1^	-	6	[82]
Urine	Diclofenac	LDH/GO/PVDF	HPLC-UV	0.14 μg L^−1^ in water 0.23 μg L^−1^ in urine 0.57 μg L^−1^ plasma samples	89–93	7 7 7	[84]
	Benzodiazepines	C18/glue	HPLC-MS	0.05−0.15 ng mL^−1^	-	5−7	[25]
	Codeine acetamiprid	Aptamer/cellulose	ESI-IMS	3.7 ng mL^−1^	87–91	2–6	[106]
	Methamphetamine	Aptamer/CDs/ cellulose	ESI-IMS	0.45 ng·mL^−1^	87–108	<8	[107]
	Codeine	Aptamer/cellulose	ESI-IMS	3.4 ng·mL^−1^	90	7	[108]
	Nonsteroidal anti-inflammatory drugs	p-PIL-AcO	LC-MS/MS.	3.8 μg L^−1^ for indomethacin 7.2 μg L^−1^ for diclofenac 6.8 μg L^−1^ for tolmetin 9.4 μg L^−1^ for ketoprofen 15.7 μg L^−1^ for naproxen 5.1 μg L^−1^ for ibuprofen	72–95	1–13	[115]
	Nonsteroidal anti-inflammatory drugs: naproxen, aspirin, tolmetin, celecoxib	MOF-5	HPLC-UV	0.57–0.77 μg L^−1^	94–108	4–6	[143]
	Endocrine-disrupting compounds	DES	LC-MS/MS	0.01–1.15 ng mL^−1^	-	3–10	[178]
	Tramadol	Ni(DMG)2-NiO-Cell	HPLC-UV	0.1–1.0 ng mL^−1^	86	6–8	[156]
	Carvedilol blocker	g-C3N4/N6 NC	FS	1.0 ng mL^−1^	83	4	[158]
	Fentanyl, methadone, zolpidem	Octyl-cyanopropyl/PAN	HPLC-MS/MS	4.0–17.4 ng mL^−1^	43–76	<15	[157]
Urine	Fluoxetine	GO/CS	HPLC-UV-Vis	1.0 ng mL^−1^	82	≤9	[152]
	Tricyclic antidepressants	Ni-Co MOFs-PAN	HPLC-UV	0.06–0.3 µg L^−1^	91–100	<5	[153]
	Biogenic monoamines	HLB/PAN	UPLC-MS/MS		36–75	<9	[179]
Plasma	Tricyclic antidepressants	MIP	LC-MS/MS	1.0–5.0 ng mL^−1^	90–110	15	[62]
	Antidepressant drugs: Clomipramine, Clozapine, Trimipramine	PVA/CA/β-cyclodextrin/Bi_2_S_3_@g-C_3_N_4_	GC-FID	0.03–0.15 ng mL^−1^	78–95	5–7	[154]
	Mycophenolic acid	MIP	UPLC	0.3 ng mL^−1^	-	4	[64]
	Benzodiazepines	C18-TEOS	LC-MS/MS	0.4–0.7 ng mL^−1^	11–83	4–8	[101]
		C18/PAN	LC-MS/MS	0.08–0.2 ng mL^−1^	83–98	<9	[44]
	Anti-cancer drugs	Co-MOF-74/polyfam	HPLC-UV	0.03–0.20 µg L^−1^	-	3–9	[71]
		polylactic acid PLA	HPLC	0.03 µg L^−1^	-	8	[180]
	Tramadol	Ni(DMG)_2_-NiO-Cell	HPLC-UV	0.1–1.0 ng mL^−1^	92	6–8	[156]
Plasma	Carvedilol	g-C_3_N_4_/N_6_ NC	FS	1.0 ng mL^−1^	87	3.6	[158]
	Fentanyl, methadone, zolpidem	Octyl-cyanopropyl/PAN	HPLC-MS/MS	4.3–8.3 ng mL^−1^	34–62	<15	[157]
	Fluoxetine	GO/CS	HPLC-UV-Vis	1.6 ng mL^−1^	87	≤9	[152]
Oral fluid	Fentanyl, methadone, zolpidem	Octyl-cyanopropyl/PAN	HPLC-MS/MS	4.8–9.6 ng mL^−1^	27–38	<15	[157]
Saliva	β-blockers	GO/PEG	LC-MS/MS	1.25–8.00 nmol L^−1^	80–109	4–13	[87]
	Methamphetamine	aptamer/CDs/cellulose	ESI-IMS	0.6 ng·mL^−1^	87–108	6	[107]
Exhaled air condensate	Aldehydes	PS/G	HPLC	3.8 nmol L^−1^	80–106	16	[86]
Skin	Volatile organic compounds	PDMS	GC-MS	-	-	<9	[37]
Fish tissue	Polychlorinated biphenyls	PDMS	LC-MS	-	-	-	[35]
	Pharmaceuticals	C18/PAN	LC/MS-MS	0.08–0.21 ng g^−1^	-	9–18	[102]
Fish plasma	Steroid hormones		LC-MS/MS	0.006–0.150 ng mL^−1^	-	≤6 ≤15	[103]

## 10. Summary

Solid-phase microextraction SPME is one of the sample preparation techniques compatible with green analytical chemistry because it reduces toxic organic solvents to the minimum necessary. TF-SPME devices, in the form of a rectangular metal or polymer substrate onto which a thin layer of sorption phase or self-supporting membranes is applied, are characterized by a higher sorption capacity. They enable faster microextraction of analytes compared to microextraction carried out on SPME fiber and can be applied in the solution of analytical problems in environmental, food, and bioanalysis. TF-SPME strips can also be used as in vivo collection devices for organic compounds emitted from human skin. In recent years, developments in drone technology have enabled the TF-SPME instrument to be used as an extraction probe to collect environmental samples in locations that are difficult to access or where their contamination levels may pose a risk to the analyst.

The active phase on which analyte sorption occurs can be applied to the substrate through techniques such as dip coating, spin coating, electrospinning, rod coating, and spray coating. The dynamic development of material chemistry enables the use of highly advanced materials as selective sorption phases in TF-SPME, such as polymers, conductive polymers, molecularly imprinted polymers, organometallic frameworks, carbon nanomaterials, aptamers, polymeric ionic liquids, and deeply eutectic solvents. The above-mentioned materials differ in their affinity to different analyte groups and consequently in their selectivity in adsorption of analytes from complex matrices. In terms of selectivity, materials such as organometallic frameworks, aptamers, molecularly imprinted polymers take the spotlight. Table 13, Table 14 and Table 15, containing detection limits and analyte recoveries in microextractions, show that the above-described materials, used as sorption phases, provide selective microextractions and sensitive determinations with detection limits at the nanograms per liter level. As a result, TF-SPME is successfully used to prepare analytical samples to determine a vast spectrum of analytes in sample environmental, biological, and food matrices.

For several years, commercial TF-SPME kits with sorption phases that enable the microextraction of polar as well as non-polar analytes have also been available and offered, among others, by GERSTEL and Markes International. The commercialization of TF-SPME devices could increase their use in analytical procedures carried out in both research and industrial laboratories in the coming years.

It can be conjectured that in the near future, the interest of researchers in developing selective and low-cost sorbents dedicated to the TF-SPME technique, as well as other solid-phase microextraction techniques, will be directed toward biopolymers and recycled materials. Even if the sorption properties of biopolymers and recycled materials are not satisfactory, a suitable research direction will be the modification of their surfaces by introducing new or additional functional groups, as well as the preparation of composite materials based on MIPs, MOFs, or CNMs.

## Figures and Tables

**Table 1:** Techniques used for preparation of TF-SPME devices.

Technique	Film Thickness Achieved	Advantages	Disadvantages
Dip coating	Low μm range	High homogeneity and mechanical stability of a layer Simple process and setup Low cost	Difficult to coat flexible substrates Large amount of solution wastage.
Spin coating	Hundreds of μm	High mechanical stability of a layer A simple and effective process that requires minimal training to master Cost-effective No requirement for any high-energy processes Possibility to produce TF-SPME extractive phase with or without a supportive core	Variation in the thickness between the central part and outer part of the prepared extractive phase High level of solution waste
Electrospinning coating	nm–μm range	High homogeneity and mechanical stability of a layer Possibility to produce TF-SPME extractive phase without a supportive core	Sensitivity to the manufacturing environment (temperature and humidity) Slow process rate
Bar coating	Hundreds of μm	Possibility to produce TF-SPME extractive phase with or without a supportive core Possibility to prepare uniform films on rigid or flexible substrates High homogeneity and mechanical stability of a layer Simplicity to change the coating thickness Simple and quick process Low cost	Thickness of layer depends on rod structure and solution properties Difficult to obtain repeatable coatings
Spray coating	μm–mm range	Simple process High mechanical stability of a layer Low cost	Poor homogeneity of the layer

**Table 2:** Examples of the use of polymer sorption phases in TF-SPME.

Sorption Phase	Analyte	Matrix	Detection	Reference
PDMS	PAHs	water	GC-MS	[34]
	polychlorinated biphenyls	fish tissue	LC-MS	[35]
	pesticides	water	DCBI-MS	[36]
	volatile organic compounds	skin	GC-MS	[37]
	insect pheromones	air	GC-MS	[38]
	drugs and explosive substances	standards in different solvents	IMS	[39]

**Table 3:** Examples of the use of polymer sorption phases in TF-SPME.

Sorption Phase	Preparation	Analyte	Matrix	Detection	Reference
DVB/PDMS	bar coating	VOCs/SVOCs	water	GC-TMS	[40]
DVB/PDMS	spin coating	N-nitrosamines	water	GC-MS	[22]
DVB/PDMS	bar coating	VOCs	water	GC-MS	[24]
DVB/PAN	ready for use	anti-inflammatory antibacterial drugs	drinking water	LC-ESI-MS/MS	[41]
DVB/PDMS	bar coating	pesticides	water	GC-MS	[42]
DVB/PDMS	bar coating	pesticides	water	GC-MS	[43]
C18/PAN	dip coating spray coating	benzodiazepines	blood plasma	LC-MS/MS	[44]
C18/PAN	spray coating	doping agents	urine, blood plasma	LC-MS	[45,46]
C18/PAN	spray coating	estrogens	water	LC-UV	[47]

**Table 4:** Examples of the use of conductive polymers in TF-SPME.

Sorption Phase	Preparation	Analyte	Matrix	Detection	Reference
PmPDA/CNT	electrospinning coating	copper	water, food	FAAS	[51]
PPy/PA	electrospinning coating	pesticides	water	GC-MS	[52]
PANI	gluing	hormones	urine	HPLC-FLD	[53]
PANI-N6	electrospinning coating	chlorobenzenes	water	GC-MS	[54]

**Table 5:** Examples of the use of MIPs in TF-SPME.

Template	Functional Monomer	Crosslinking Reagent	Analyte	Matrix	Detection	Ref.
2-thiophenocarboxyaldehyde	1-vinylimidazole	bisphenol dimethacrylate	PASHs	seawater	GC–MS	[59]
2-{[diethoxy(sulfanylidene)-λ-phosphanyl]amino}acetic acid	methacrylic acid	ethylene glycol dimethacrylate	OPPs	water	LC-MS/MS	[60]
catechol	4-vinyl benzoic acid	ethylene glycol dimethacrylate	phenols	water	UHPLC-PDA	[61]
(3-(10,11-dihydro-5H-dibenzo[b,f]azepin-5-yl)propyl)(methyl) carbamate	methacrylic acid	ethylene glycol dimethacrylate	TCAs	plasma	LC-MS/MS	[62]
phenol	itaconic acid, 4-vinylpyridine, styrene	ethylene glycol, dimethacrylate, triethylene glycol dimethacrylate, pentaerythritol triacrylate	phenols	water	LC-MS	[63]
mycophenolate mofetil	4-vinylpyridine	ethylene glycol dimethacrylate	MPA	plasma	UPLC	[64]

**Table 6:** Examples of the use of MOFs in TF-SPME.

Sorption Phase	Preparation	Analyte	Matrix	Detection	Reference
DUT-52/PVDF	bar coating	PCPs	cosmetics	UHPLC-UV/VIS	[70]
Co-MOF-74/polyfam	electrospinning coating	anti-cancer drugs	water sewage, plasma	HPLC-UV	[71]
MIL-53(Al)/PVDF MIL-53(Fe)/PVDF MIL-100(Fe)/PVDF MIL-101(Cr)/PVDF UiO-66(Zr)/PVDF	bar coating	estrogens	urine	HPLC-FLD	[72]
ZIF-8/LDH/GO/PVDF	bar coating	caffeine	urine	HPLC-UV	[73]
amino-Zr-MOF/PAN	electrospinning coating	pesticides	water	CD-IMS	[74]
MIL-100(Fe)/PS/cellulose	dip coating	PCPs	pool water, cosmetics	HPLC-PDA	[75]
MOF-199/PS	electrospinning coating	aldehydes	urine	HPLC-VWD	[76]

**Table 7:** Examples of the use of graphene materials in TF-SPME.

Sorption Phase	Preparation	Analyte	Matrix	Detection	Ref.
CY-GO-LDH	dip coating	non-steroidal anti-inflammatory drugs	plasma, urine	HPLC-UV	[82]
graphene membrane	drop casting	metal ions	water	TXRF	[83]
LDH/GO/PVDF	application to a Petri dish	diclofenac	urine	HPLC-UV	[84]
GO	ESD	metal ions	water	LIBS	[85]
PS/G	electrospinning coating	aldehydes	exhaled air condensate	HPLC	[86]
GO/PEG	dip coating	β-blockers	saliva	LC-MS/MS	[87]
ZIF-8@GO	dip coating	macrolides, lincosamides	honey	UPLC-MS/MS	[88]
PAMAM@GO-PVDF	application to a Petri dish	OPPs	water	HPLC-UV	[89]

**Table 8:** Examples of the use of carbon nanotubes in TF-SPME.

Sorption Phase	Preparation	Analyte	Matrix	Detection	Ref.
SDS-MWCNTs/PP	application on PP substrate	glucocorticoids	water	UHPLC-MS	[93]
MWCNT/Agr-Ch	application to a Petri dish	non-steroidal anti-inflammatory drugs	water	HPLC-UV	[94]
MWCNTs/Agr	application to a Petri dish	PAHs	green tea drink	HPLC-UV	[95]
MWCNTs/CTA	application to a Petri dish	fungicides personal care products PCPs	water	GC-MS	[96]
MWCNTs- COOH/PDMS	dip coating	phenolic compounds	water	HPLC-UV	[97]
Agr-Ch-MWCNTs	application to a Petri dish	TCAs	water	HPLC-UV-Vis	[98]
MWCNTs-COOH-Ch/PP	dip coating	PCBs	water	GC-MS	[99]
MWCNTs/CTA	application to a Petri dish	PAHs	water	HPLC-UV	[100]

**Table 9:** Examples of the use of C18 silica in TF-SPME.

Sorption Phase	Preparation	Analyte	Matrix	Detection	Reference
C18-TEOS	dip coating	benzodiazepines	plasma	LC–MS/MS	[101]
C18/PAN	spray coating	pharmaceuticals	fish tissue	LC/MS–MS	[102]
C18/PAN	spray coating	steroid hormones	fish plasma	LC–MS/MS	[103]
C18/SCX	commercial strips	carbamazepine triclosan	water	DESI-MS	[104]
C18/glue	dip coating	benzodiazepines	urine	HPLC-MS	[25]

**Table 10:** Examples of the use of aptamers in TF-SPME.

Sorption Phase	Preparation	Analyte	Matrix	Detection	Reference
aptamer/cellulose	dip coating	codeine acetamiprid	urine, vegetable juice water	ESI-IMS	[106]
aptamer/CDs/cellulose	dip coating	methamphetamine	urine, plasma saliva	ESI-IMS	[107]
aptamer/cellulose	dip coating	codeine	urine	ESI-IMS	[108]

**Table 11:** Examples of the use of polymeric ionic liquids in TF-SPME.

Sorption Phase	Preparation	Analyte	Matrix	Detection	Reference
HVImBr/MMA	application to the Petri dish	sulfathiazole	milk, honey	SF	[114]
p-PIL-AcO	dip coating	nonsteroidal anti-inflammatory drugs	urine	LC-MS/MS.	[115]
p-Poly-(MMA-IL)	dip coating	sulfonamides	water	HPLC-DAD	[116]
poly-(MMA-BVImBr)	application to the Petri dish	aniline	water	LC-MS/MS	[117]

**Table 12:** Examples of the Use of DES in TF-SPME.

Sorption Phase	Preparation	Analyte	Matrix	Detection	Ref.
DES	dip coating	parabens	water	HPLC-UV	[119]
	dip coating	formaldehyde	water	HPLC-UV-Vis	[122]
PDMS/DES	dip coating	parabens	water	HPLC-UV	[121]

## Abbreviations

AAS	Atomic absorption spectroscopy
ACGO	Graphene oxide-coated agarose/chitosan
AES	Atomic emission spectroscopy
Agr	Agarose
AV	Aloe vera gel
BVImBr	1-butyl-3-vinylimidazolium bromide
CA	Citric acid
CA	Citric acid
CC	Carbon cloth
CD-IMS	Corona discharge ion mobility spectrometry
CDs	Carbon dots
Ch	Chitosan
CNMs	Carbon nanomaterials
CNT	Carbon nanotube
CPs	Conductive polymers
CS	Chitosan
CTA	Cellulose triacetate
CVD	Chemical vapor deposition
CY	Cotton yarn
CY-GO-LDH	Cotton yarn–graphene oxide-layered double hydroxide composite
DAD	Diode array detector
DBS	Dibutyl sebacate
DCBI-MS	Desorption corona beam ionization mass spectrometry
DES	Deep eutectic solvents
DESI-MS	Desorption electrospray ionization mass spectrometry
DI-SDME	Direct immersion single-drop microextraction
DLLME	Dispersive liquid–liquid microextraction
DNA	Deoxyribonucleic acid
ESD	Electrospray deposition technique
ESI-IMS	Electrospray ionization ion mobility spectrometer
FAAS	Flame atomic absorption spectroscopy
FID	Flame ionization detector
FMSNs	Fibrous mesoporous silica nanospheres
FS	Fluorescence spectrometry
G	Graphene
GBMs	Graphene-based materials
GC	Gas chromatography
GC-MS	Gas chromatography–mass spectrometry
GC-TMS	Gas chromatography–toroidal ion trap mass spectrometry
GO	Graphene oxide
HBD	Hydrogen bond donor
HF-LPME	Hollow-fiber liquid phase microextraction
HLB	Hydrophilic–lipophilic balance
HPLC	High-performance liquid chromatography
HPLC-VWD	High-performance liquid chromatography system with a variable wavelength ultraviolet detector
HPLC-DAD	High-performance liquid chromatography system with a diode array detector
HPLC-FLD	High-performance liquid chromatography with fluorescence detection
HPLC-MS	High-performance liquid chromatography with mass spectrometry
HPLC-PDA	High-performance liquid chromatography with photodiode array detector;
HPLC-UV	High-performance liquid chromatography with ultraviolet spectrophotometry
HPLC-UV-Vis	High-performance liquid chromatography with ultraviolet–visible spectrophotometry
HS-GC-SCD	Headspace gas chromatography sulfur chemiluminescence detection
HS-SDME	Headspace single-drop microextraction
HVImBr	1-hexyl-3-vinylimidazolium bromide
ILs	Ionic liquids
IMS	Ion mobility spectrometry
LC/MS–MS	Liquid chromatography–tandem mass spectrometry
LC-ESI-MS/MS	Liquid chromatography/electrospray ionization–tandem mass spectrometry
LC-MS	Liquid chromatography and mass spectrometry
LC-UV	Liquid chromatography with ultraviolet spectrophotometry
LIBS	Laser-induced breakdown spectroscopy
LLE	Liquid–liquid extraction
LOD	Limit of detection
LOQ	Limit of quantification
LPME	Liquid-phase microextraction
MALDI-TOF MS	Matrix-assisted laser desorption/ionization time-of-flight mass spectrometry
MEPS	Microextraction in packed syringe
METs	Microextraction techniques
MIPs	Molecularly imprinted polymers
MMA	Methylmethacrylate
MOFs	Metal–organic frameworks
MPA	Mycophenolic acid
MWCNT	Multi-wall carbon nanotubes
NPOE	Nitrophenyl octyl ether
OCTNs	Oxidized carbon nanotubes
OPPs	Organophosphorus pesticides
PA	Polyacetylene
PAMAM	Poly(amidoamine)
PAN	Polyacrylonitrile
PANI	Polyaniline
PASHs	Polycyclic aromatic sulfur heterocycles
PCBs	Polychlorinated biphenyls
PCN	Polycyanamide
PCPs	Personal care products
PDMS	Polydimethylsiloxane
PEDOT	Polyethylenedioxytiofene
PEG	Polyethylene glycol
PES	Polyether sulfone
PF	Polyfluorene
PFu	Polyfuran
PILs	Poly(ionic liquids)
PLOT	Porous layer open tubular
PMMA	Polymethyl methacrylate
PMME	Polymer monolith microextraction
PP	Polypropylene
PPA	Polyphenylacetylene
p-PIL-AcO	Pil-coated paper with acetate
p-Poly-(MMA-IL)FP	Paper-based polymeric ionic liquid
PPP	Polyparaphenylene
PPV	Polyphenylenevinylene
PPy	Polypyrrole
PPY	Polypyridine
PS	Polystyrene
PTFE AF	Polytetrafluoroethylene amorphous fluoropolymer
PTh	Polythiophene
PU	Polyurethane
PVA	Polyvinyl alcohol
PVA/MCS/HC-POF	Polyvinyl alcohol/modified chitosan/hydroxy-containing porous organic framework
PVA-SA-βCD	Polyvinyl alcohol doped with beta cyclodextrin and alginate
PVDF	Poly(vinylidene fluoride)
pVFc	Polyvinylferrocene
PVP	Polyvinyl pyrrolidone
rGO	Reduced graphene oxide
RNA	Ribonucleic acid
SBSE	Stir bar sorptive extraction
SBU	Secondary building units
SCOT	Support-coated open tubular
SDME	Single-drop microextraction
SDS	Sodium dodecyl sulfate
SELEX	Systematic evolution of ligands by exponential enrichment
SEM	Scanning electron microscope
SERS	Surface-enhanced Raman scattering
SF	Spectrofluorometry
SPDE	Solid-phase dynamic extraction
SPE	Solid-phase extraction
SPME	Solid-phase microextraction
SVOCs	Semi-volatile organic compounds
SWCNT	Single-wall carbon nanotubes
TCAs	Tricyclic antidepressant drugs
TEM	Transmission electron microscope
TEOS	Tetraethoxysilane
TFME	Thin-film microextraction
TXRF	Total reflection X-ray fluorescence spectrometry
UHPLC-MS	Ultra-high-performance liquid chromatography with mass spectrometry
UHPLC-PDA	Ultra-high-performance liquid chromatography with photodiode array detector
UHPLC-UV/VIS	Ultra-high-performance liquid chromatography with ultraviolet–visible spectrophotometry
UPLC	Ultra-performance liquid chromatography
UPLC–MS/MS	Ultra-performance liquid chromatography with tandem mass spectrometry
VOCs	Volatile organic compounds
WA	Aliphatic hydrocarbons
WCOT	Wall-coated open tubular
WWA	Polycyclic aromatic hydrocarbons
ZIFs	Zeolitic imidazolate frameworks
